# UGA codon position-dependent incorporation of selenocysteine into mammalian selenoproteins

**DOI:** 10.1093/nar/gkt409

**Published:** 2013-05-28

**Authors:** Anton A. Turanov, Alexei V. Lobanov, Dolph L. Hatfield, Vadim N. Gladyshev

**Affiliations:** ^1^Division of Genetics, Department of Medicine, Brigham and Women’s Hospital and Harvard Medical School, Boston MA 02115, USA and ^2^Molecular Biology of Selenium Section, Laboratory of Cancer Prevention, Center for Cancer Research, National Cancer Institute, National Institutes of Health, Bethesda, MD 20892, USA

## Abstract

It is thought that the SelenoCysteine Insertion Sequence (SECIS) element and UGA codon are sufficient for selenocysteine (Sec) insertion. However, we found that UGA supported Sec insertion only at its natural position or in its close proximity in mammalian thioredoxin reductase 1 (TR1). In contrast, Sec could be inserted at any tested position in mammalian TR3. Replacement of the 3′-UTR of TR3 with the corresponding segment of a *Euplotes crassus* TR restricted Sec insertion into the C-terminal region, whereas the 3′-UTR of TR3 conferred unrestricted Sec insertion into *E. crassus* TR, in which Sec insertion is normally limited to the C-terminal region. Exchanges of 3′-UTRs between mammalian TR1 and *E. crassus* TR had no effect, as both proteins restricted Sec insertion. We further found that these effects could be explained by the use of selenoprotein-specific SECIS elements. Examination of Sec insertion into other selenoproteins was consistent with this model. The data indicate that mammals evolved the ability to limit Sec insertion into natural positions within selenoproteins, but do so in a selenoprotein-specific manner, and that this process is controlled by the SECIS element in the 3′-UTR.

## INTRODUCTION

Mammalian selenoproteins are a diverse group of proteins, which contain selenocysteine (Sec), which is known as the 21st genetically encoded amino acid (1,2). Humans have 25 selenoprotein genes, and the biological functions of these selenoproteins strictly depend on the Sec residue, which is usually located at the enzyme active sites and serves a catalytic redox-active function (3). Incorporation of selenium as Sec into selenoproteins occurs via a specific mechanism that recodes the UGA codon from its normal translation termination function. Sec biosynthesis and incorporation also depends on several proteins, including selenophosphate synthetase 2, which converts selenide to monoselenophosphate and is itself a selenoprotein, phosphoseryl-tRNA^(Ser)Sec^ kinase that phosphorylates the seryl moiety on seryl-tRNA^(Ser)Sec^, Sec synthase that synthesizes Sec on its tRNA, a Sec-specific elongation factor, SelenoCysteine Insertion Sequence (SECIS)-binding protein 2 (SBP2), ribosomal protein L30 and possibly several other factors (4,5). The selenoprotein synthesis machinery also uses a specific structure in the 3′-UTRs of selenoprotein mRNAs, termed the SECIS element that recruits protein factors and Sec tRNA to synthesize Sec and incorporate it into nascent polypeptides in response to UGA codons (6,7). Despite great progress in our understanding of Sec biosynthesis and incorporation into proteins, many of the detailed mechanisms that regulate Sec insertion remain to be investigated.

In mammals and other eukaryotes, Sec insertion at in-frame UGA codons requires the presence of SECIS elements in the 3′-UTRs (8). The Sec encoding UGA codons may occur in various locations within genes (i.e. N-terminal, middle and C-terminal regions), and SECIS elements may be present at different distances from the Sec codon, stop signal and polyA tail (9,10). There is only one mammalian selenoprotein with more than one Sec, selenoprotein P (SelP, SEPP1) (11). Human SelP has 10 Sec residues, which are encoded by 10 in-frame UGA codons, and the protein also has two SECIS elements in its 3′-UTR. Thus, the presence of a single Sec requires a single SECIS element in the 3′-UTR, whereas to insert multiple Sec residues, two SECIS elements may be needed. It is unclear, however, what determines the efficiency of Sec insertion: UGA position within the coding sequence, position of the SECIS element in the 3′-UTR, structure and type of SECIS element, overall structure of selenoprotein mRNA, a combination of these factors and/or additional factors.

We previously examined Sec insertion into *Euplotes crassus* thioredoxin reductases (TRs) (12). In this ciliate, UGA codes for both Sec and Cys (but it is not used for termination of translation), and several of its selenoproteins contain multiple UGA codons that can code unambiguously for Sec or Cys. Interestingly, Sec insertion into *E. crassus* TR1 was strictly dependent on the position of UGA within its coding sequence (12). Whether other organisms restrict Sec insertion to specific positions within their coding sequences is not known. It was previously shown that, in mouse glutathione peroxidase 1, Sec can be inserted in different positions in the protein (13). Although efficiency of Sec insertion in glutathione peroxidase 1 somewhat differed depending on the location of the UGA codon, these findings appeared to be different from what was observed in *Euplotes*.

Methods that are often used to assess efficiency of Sec insertion into proteins are based on UGA codon read-through analyses, e.g. using luciferase activity assays (10,14–16). These analyses led to important insights into Sec incorporation mechanisms, but they could not completely reproduce Sec insertion into endogenous selenoproteins. In this regard, metabolic labeling of cells with ^75^Se is a well-established procedure to monitor Sec incorporation into natural selenoproteins. In the present study, we used this method to investigate SECIS element- and UGA position-dependent Sec insertion into mammalian selenoproteins.

## MATERIALS AND METHODS

### Bioinformatics analyses

**Figure 1.**
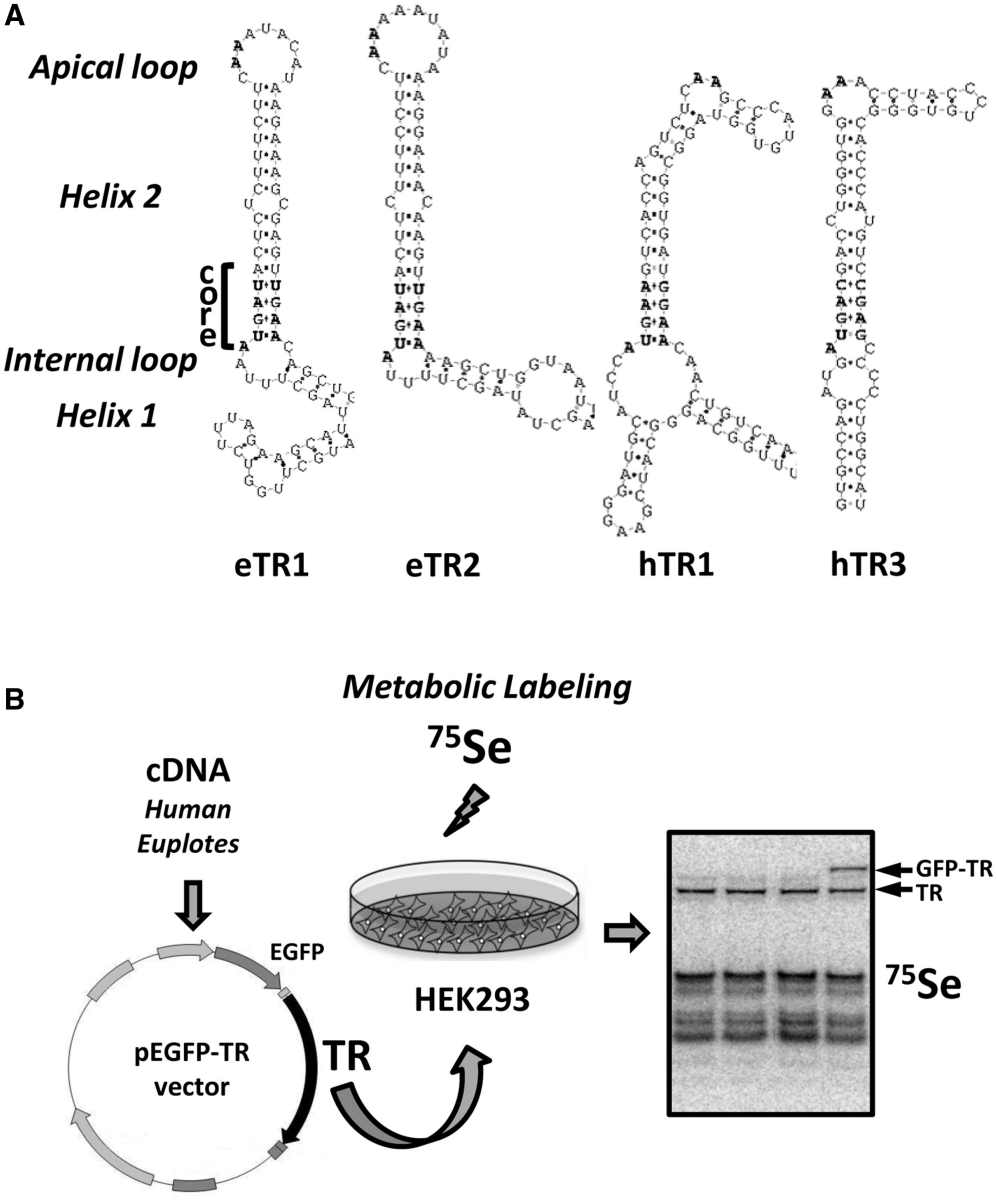
Experimental design of the study. (**A**) SECIS elements used in the study. SECIS element images were generated with *SECISearch*. The SECIS element core and apical loop are shown in bold, and the essential structural motifs are highlighted. Type II SECISes contain an additional stem in the apical loop. eTR1 and eTR2 designate *Euplotes* TR1 and TR2, respectively, and hTR1 and hTR3 designate human TR1 and TR3. (**B**) Design of the study. cDNAs corresponding to selenoproteins are cloned into the pEGFP-C3 vector (unless indicated otherwise). Site-directed mutagenesis is used to introduce mutations in the original sequences. HEK 293 cells are transfected with the resulting constructs, and 24 h after transfection, cells are labeled by supplementing the medium with ^75^Se for an additional 24 h. Proteins from each transfection are resolved by SDS–PAGE, and selenoprotein patterns are visualized. The EGFP-fusion of TRs is used to distinguish the expressed proteins (e.g. EGFP-TR; shown with an arrow) from endogenous selenoproteins (e.g. TR, shown with an arrow). Other detected bands represent endogenous selenoproteins, which serve as an internal control.

Natural and mutant SECIS elements were analyzed with SECISearch (1,2,17). Structures of SECIS elements used in this study are shown in Figure 1A. Other sequence analyses were done with BLAST programs. The minimum free energy for secondary structures of SECIS elements were calculated using Vienna RNA package (http://rna.tbi.univie.ac.at/cgi-bin/RNAfold.cgi).

### Constructs and cloning

Standard cloning procedures were used to prepare natural, chimeric and other mutant forms of selenoprotein genes and SECIS elements. Full-length human thioredoxin reductase 1 (hTR1, Txnrd1) (BC018122) cDNA and the cDNA coding for mature (without its mitochondrial signal) thioredoxin reductase 3 (hTR3, Txnrd2) (NM_006440), including their 3′-UTRs, were amplified by PCR from a HEK 293T cDNA library with sequence specific primers and Pfu Ultra DNA polymerase (Agilent) as described (18,19) and cloned into the pEGFP-C3 vector. Chimeric human-*Euplotes* (human TR ORFs with *Euplotes* TR 3′-UTRs also containing sequences coding for the C-terminal 20 amino acids) as well as *Euplotes*-human fusion sequences were introduced by a two-step cloning procedure. Replacements of SECIS elements were done by cloning fragments, in which SECIS element sequences were introduced using specific primers. pcDNA 3.1 (Promega) was used for cloning thioredoxin-glutathione reductase (mTGR, Txnrd3) (NM_153162) from a mouse testis cDNA library as described (20). Human c-myc tag (decapeptide sequence EQKLISEEDL) was also introduced resulting in an N-terminally-tagged mTGR construct, and the Kozak consensus sequence (5′-CCACC**ATG**G-3′) was added to maximize translation efficiency of c-myc-mTGR in eukaryotic cells (21). *E. crassus* TR1 and TR2 (eTR1 and eTR2, respectively) as well as selenoprotein W3 (eSelW) were cloned into pEGFP-C3 as described previously using the *E. crassus* macronuclear DNA as a template (12). To extend the distance between the Sec-encoding UGA codon and the SECIS element core in eTR2 two fold (2 × eTR2), the corresponding region was amplified by PCR and then cloned in the initial eTR2 construct. A c-myc tag with the Kozak consensus was introduced to the eSelW construct and cloned into pCI-neo. Sequence of a segment of the 3′-UTR from human selenoprotein P (Sepp1) (BC015875) containing two SECIS elements was amplified from a cDNA (clone id:4715833; MHS1011-9199101, Open Biosystem/Thermo). The resulting fragment was cloned into pEGFP-C3 containing eTR2 ORF. QuikChange™ Site-Directed Mutagenesis Kit (Agilent) was used to generate mutations at in-frame UGA codons in *Euplotes* selenoprotein genes, or introduce unnatural UGA codons at indicated positions in human genes. Site-directed mutagenesis was also used to delete 8 or 11 nt from the apical loop of hTR3 SECIS. All mutations were verified by sequencing. Nucleotide sequences for all used constructs are shown in the Supporting Material (Supplementary Figures S1–S18).

### Cell culture and transfection

HEK 293T cells (ATCC), cultured in Dulbecco’s modified Eagle’s medium supplemented with 10% FBS, 100 U/ml penicillin and 100 units/ml streptomycin (Invitrogen) were transfected with the resulting constructs using a standard calcium phosphate method or with CalPhos calcium phosphate mammalian cell transfection kit (Clontech) (12). Cells were 40–60% confluent at the time of transfection. Efficiency of transfection was above 50% (based on the fraction of EGFP-expressing cells).

### RNA isolation, cDNA synthesis and real-time quantitative PCR

For quantitative PCR (qPCR) experiments, HEK cells in 6-well plates were transfected with the plasmids as described earlier in the text. RNA from the cells collected 48 h after transfection was extracted using iScript RT-qPCR Sample Preparation Reagent (Bio-Rad), and cDNA was prepared with iScript cDNA Synthesis Kit (Bio-Rad) using 2 µl of total RNA. qPCR was performed using iQ SYBR Green Supermix (Bio-Rad) with 1 µl of cDNA in Bio-Rad CFX96 real-time PCR cycler. Cycling conditions were used as recommend by the manufacturer. EGFP was used as a target sequence for measuring the mRNA levels of the fusion human and *Euplotes* constructs. Glyceraldehyde-3-phosphate dehydrogenase was used as an internal standard for normalization. Oligonucleotide primers used in qPCR along with the predicted product size are listed in Supplementary Table S1 in the Supporting Material.

### ^75^Se metabolic labeling

HEK 293T cells were metabolically labeled essentially as described (12). Briefly, 24 h after transfection, cells on 10 cm plates were labeled by supplementing the medium with 50 µCi of freshly prepared [neutralized with NaOH and titrated to neutral pH with 1 M Tris–HCl (pH 8.0)] ^75^Se [(^75^Se)selenious acid (specific activity 1000 Ci/mmol; Research Reactor Facility, University of Missouri, Columbia, MO] for an additional 24 h. Cells were collected, resuspended in PBS and sonicated. In all, 30 µg of total soluble protein from each transfection were resolved by SDS–PAGE and transferred onto a PVDF membrane. Selenoproteins were visualized with a PhosphorImager. The schematic diagram of Sec insertion analysis is shown in Figure 1B.

## RESULTS

### UGA position-dependent Sec insertion into mammalian selenoproteins

**Figure 2.**
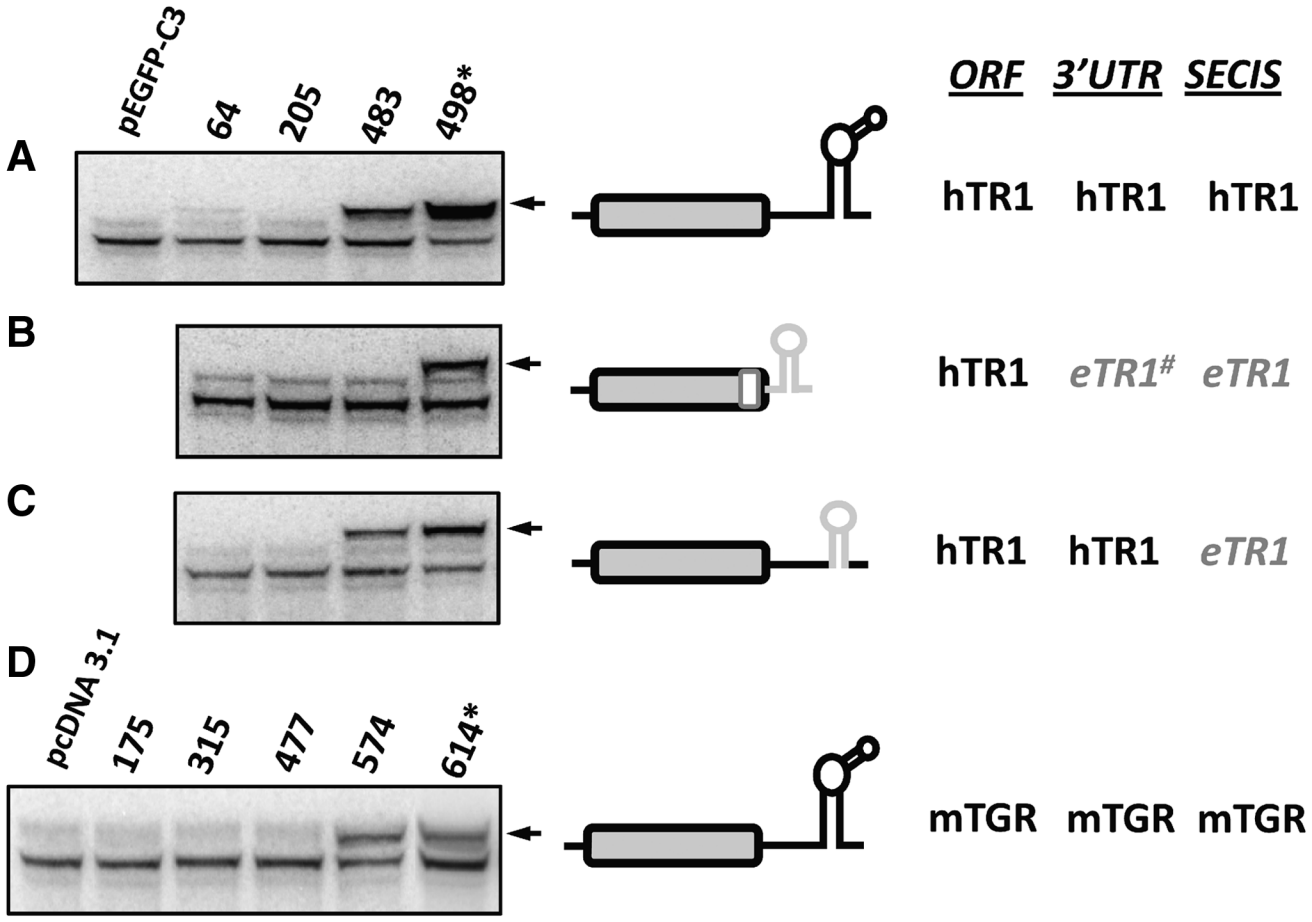
Position-dependent Sec insertion into hTR1 and mTGR. Cells were transfected with EGFP-hTR1 (**A–C**) or mTGR (**D**) constructs or with the indicated chimeric constructs containing *Euplotes* sequences. All expressed TRs had single in-frame UGA codons at indicated positions (natural or unnatural). Transfected HEK 293 cells were metabolically labeled with ^75^Se, and proteins separated by SDS–PAGE, transferred onto PVDF membranes, and selenoprotein patterns were visualized with a PhosphorImager. Left panels: Sec incorporation in TRs assayed by ^75^Se labeling. Numbers correspond to the UGA codon positions. Asterisk indicates natural positions of Sec-encoding UGA codons in TRs. Arrows show positions of full length Sec containing proteins. Middle panels: Schematic representation of constructs. Natural SECIS element sequences are shown in black, and when replaced with a foreign sequence, in gray. Right panels: This part of the figure shows ORF, 3′-UTR and SECIS element sequences that were used in indicated constructs. Hash mark indicates that the replaced sequence includes the C-terminal part of the coding sequence together with the 3′-UTR. (A) Expression of EGFP-hTR1. Sec insertion in hTR1 with its natural SECIS element. (B) Sec insertion in hTR1 with the 3′UTR from eTR1. (C) Sec insertion in hTR1 with the SECIS element from eTR1. (D) Expression of mTGR. Sec insertion in mTGR with its natural SECIS element.

To test whether human selenoproteins exhibit position-dependent Sec insertion, we prepared a set of hTR1 (Txnrd1) constructs containing single in-frame UGA codons at various positions within the coding sequence. These constructs coded for EGFP-fused hTR1. This fusion shifted migration of the selenoprotein on gels, thereby distinguishing it from selenoproteins naturally expressed in mammalian cells (schematically shown in Figure 1B). An analysis of TR1 expression from these constructs, as revealed by metabolic ^75^Se labeling of recombinant TR1 and endogenous selenoproteins in transfected HEK 293 cells, showed that Sec could be inserted at codon 498 (the natural Sec position in TR1) as well as at codon 483, but incorporation was severely decreased upstream of the C-terminal region (i.e. not detected at all at codon 205 and only weakly detected at codon 64) (Figure 2A). These findings suggested that Sec insertion may be limited to designated positions within the TR1 ORF, and more specifically to the natural Sec position and its close proximity. Replacement of the hTR1 SECIS element or the entire 3′-UTR, together with the sequence coding for the last 20 amino acids, with the corresponding sequences derived from eTR1 did not affect the observed position-dependent Sec insertion (Figure 2B and C). Moreover, if the entire 3′-UTR, together with the sequence coding for the C-terminal region, were replaced, the Sec insertion was further restricted, i.e. position 483 no longer supported Sec insertion. A qPCR analysis showed no significant differences in the corresponding mRNA levels of EGFP-hTR1 expression constructs, suggesting that the SECIS-dependent Sec insertion was regulated at the translational level (Supplementary Figure S19).

We further tested another mammalian TR, mouse TGR (Txnrd3), but in this case, EGFP was not fused, as migration of the protein on gels could be distinguished from endogenous proteins. The analysis of TGR revealed that it resembled hTR1 and *E. crassus* enzymes in that Sec could only be inserted into its natural position as well as immediately upstream of it (Figure 2D). Thus, mammalian selenoproteins are subject to the position-dependent Sec insertion.

### UGA codon position-dependent Sec insertion occurs in a selenoprotein-specific manner

**Figure 3.**
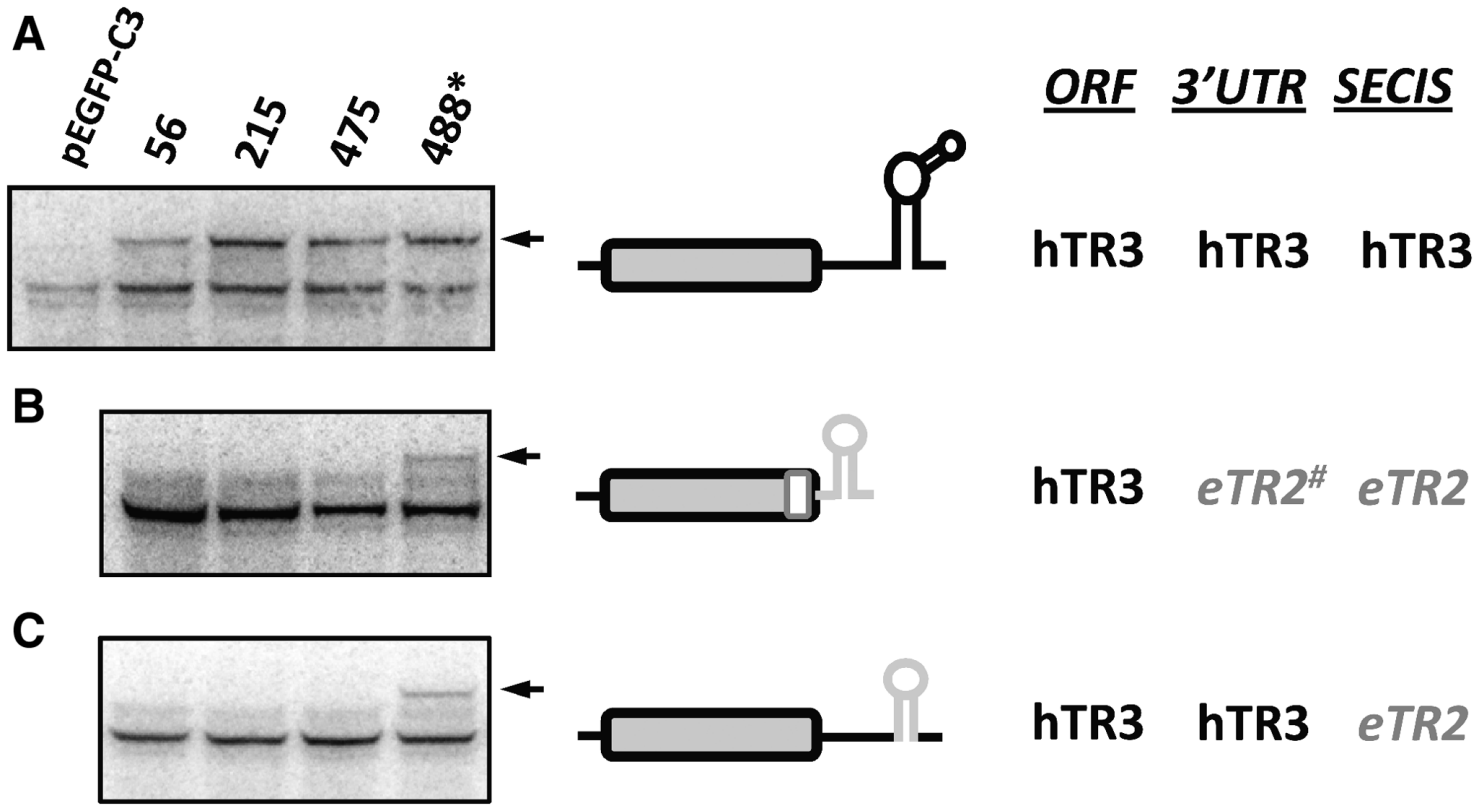
Position-dependent Sec insertion in hTR3. (**A**) Expression of EGFP-hTR3 in HEK 293 cells. Sec insertion in hTR3 with its natural SECIS element. (**B**) Sec insertion in hTR3 with the 3′UTR from eTR2. (**C**) Sec insertion in hTR3 with the SECIS element from eTR2. The experiment was carried out as described in Figure 2.

In contrast to hTR1 and mTGR, an analysis of a similar set of constructs coding for hTR3 (Txnrd2) revealed that Sec could be inserted into any tested coding position and that the insertion had similar efficiency (Figure 3A). Thus, in human cells, Sec insertion can be both position-dependent and selenoprotein-specific.

Replacement of the entire 3′-UTR of hTR3, together with the sequence coding for the last 20 amino acids, or only the SECIS element, with those of eTR2, restricted Sec insertion: in the resulting chimeric proteins, Sec could only be inserted into the natural position in the C-terminal dipeptide of the protein, similar to *Euplotes* TR1 (Figure 3B and C). As in the case of hTR1, hTR3 mRNA levels were similar for all used constructs (Supplementary Figure S20). These data suggest that the SECIS element and/or other unidentified elements in selenoprotein mRNA (e.g. mRNA structure, mRNA length) are responsible for blocking (or not able to support) Sec insertion into positions away from the natural UGA codon site.

### SECIS element defines UGA position-dependent Sec insertion

**Figure 4.**
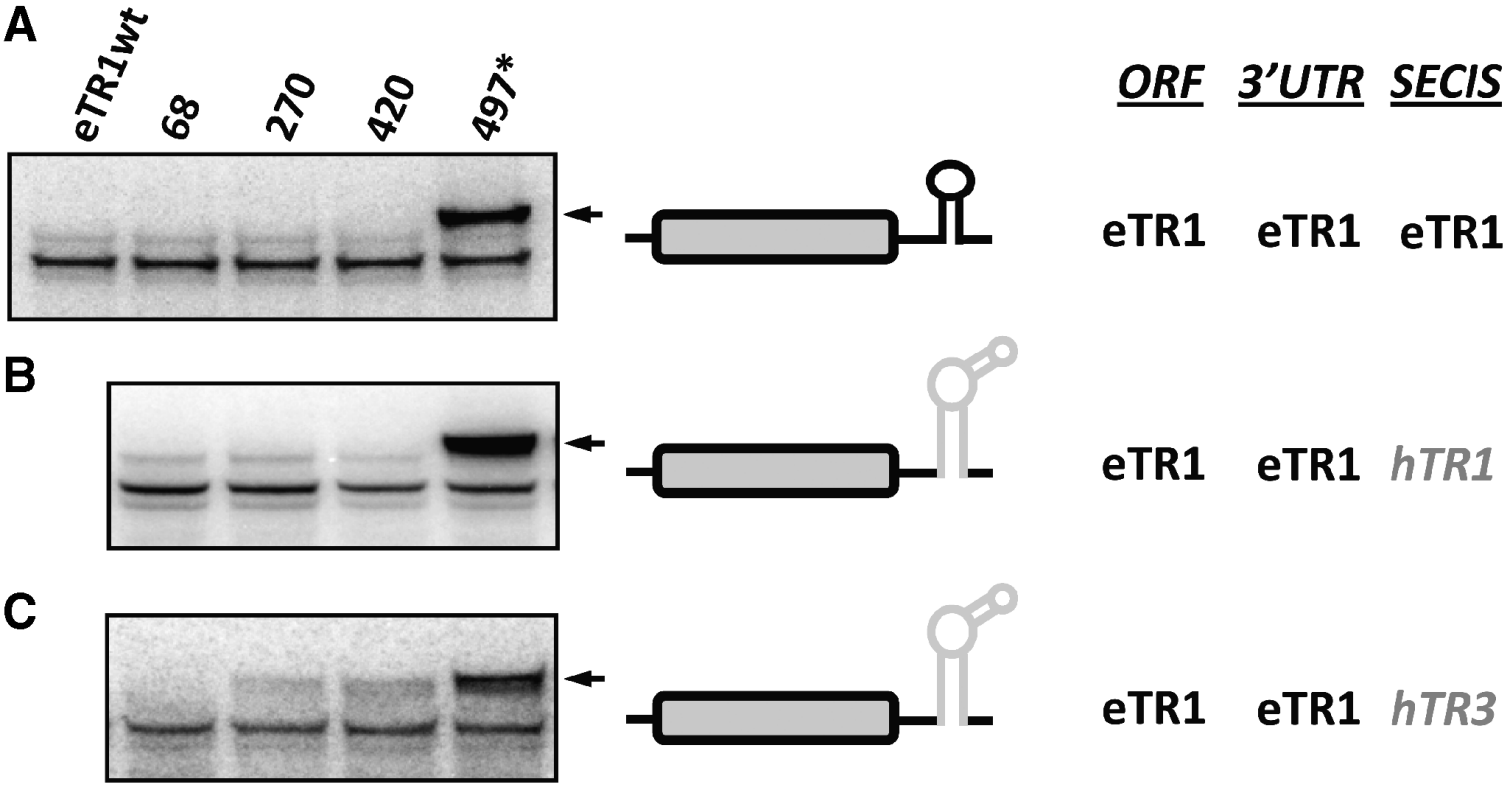
Role of SECIS elements in position-dependent Sec insertion. (**A**) Expression of EGFP-eTR1 in HEK 293 cells. Sec insertion in eTR1 with its natural SECIS element. (**B**) Sec insertion in eTR1 with the SECIS element from hTR1. (**C**) Sec insertion in eTR1 with the SECIS element from hTR3. The experiment was carried out as described in Figure 2.

The eTR1 contains seven in-frame UGA codons, with one (in position 497) coding for Sec and the other six coding for Cys in *Euplotes*. However, when this selenoprotein was expressed in mammalian cells, the Cys UGA codons specified stop signals and the very first UGA terminated protein synthesis. Thus, no Sec insertion was observed by ^75^Se labeling when the original protein with 7 UGA codons was expressed (Figure 4A). However, when the constructs were used that contained single UGA codons, Sec insertion was observed, but only in the natural Sec position (Figure 4A). As the eTR1 SECIS element supports Sec insertion only in the last 20 codons of the ORF, we used this model protein to provide insights into the mechanisms responsible for restricting Sec insertion. The major difference between *Euplotes* and human TRs is the type of SECIS element in these selenoproteins. *E. crassus* TR mRNAs possess Type I, whereas human TR mRNAs have Type II SECIS elements. First, we tested how human Type II SECIS elements function in eTR1 by replacing the eTR1 SECIS element with either hTR1 or hTR3 SECIS elements (Figure 1A). The hTR1 SECIS element only supported insertion of Sec at position 497, whereas the hTR3 structure supported Sec insertion in any position within eTR1. With the hTR3 SECIS element, Sec was efficiently inserted in eTR1 at both tested unnatural positions, 420 and 270 (Figure 4B and C).

**Figure 5.**
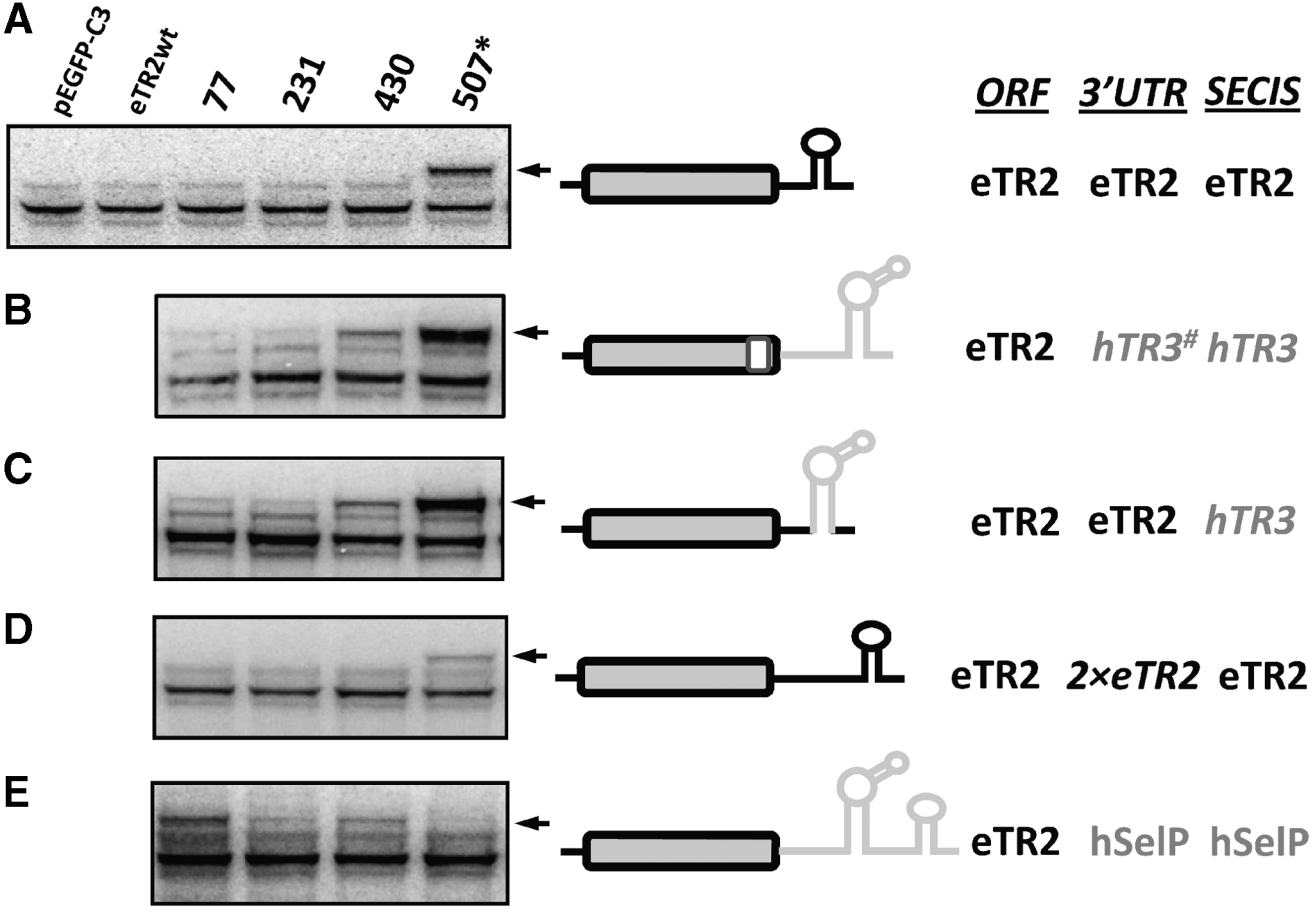
Role of the 3′-UTR and SECIS element in position-dependent Sec insertion. (**A**) Expression of EGFP-eTR2 in HEK 293 cells. Sec insertion in eTR2 with its natural SECIS element. (**B**) Sec insertion in eTR2 with the 3′-UTR from hTR3. (**C**) Sec insertion in eTR2 with the SECIS element from hTR3. (**D**) Sec insertion in eTR2 with a modified form of the natural SECIS element. (**E**) Sec insertion in eTR2 with the 3′-UTR from human selenoprotein P. The experiment was carried out as described in Figure 2. The 2 × eTR2 indicates duplication of the sequence between the natural Sec UGA codon and the SECIS core in eTR2 mRNA.

*Euplotes crassus* has another TR, eTR2, which also has seven in-frame UGA codons (but not at the same positions as in eTR1), the first six coding for Cys and the last, at position 507, coding for Sec. As was found in eTR1, Sec was only inserted in the C-terminal region of eTR2 (Figure 5A). To change the coding function of UGA codon in eTR2, we replaced its SECIS element or the entire 3′-UTR together with the part coding for the 20 C-terminal amino acids with the corresponding hTR3 region. In each case, the hTR3 SECIS element reprogrammed Sec insertion, wherein this residue could be incorporated in previously restricted positions, i.e. positions 77, 231 and 430 (Figure 5B and C).

In human TRs, the distance between stop codon and SECIS core is ∼225 nt (221 nt in hTR1 and 228 nt in hTR3), whereas in *E. crassus* this distance is shorter (138 nt in eTR1 and 124 nt in eTR2). It was previously reported that a minimum of 51 nt was required for Sec insertion, and that the distance of 200 nt or greater was needed to support mRNA flexibility for optimal SECIS function (6,13). Thus, it was possible that the shorter distance between UGA and SECIS element in eTRs was responsible for restricting Sec insertion. To test this possibility, we extended the distance between the natural UGA codon and the SECIS element in eTR2 2-fold (2 × eTR2) so that it would resemble those of hTRs, but found that this manipulation did not change the Sec coding function (Figure 5D). Additionally, we replaced the eTR2 3′-UTR with the human SelP 3′-UTR containing two SECIS elements, one of which is the Type I and another Type II SECIS elements. In this construct, Sec could be inserted at any position (Figure 5E). Sec insertion into the C-terminal region was less efficient than that in the N-terminal region.

**Figure 6.**
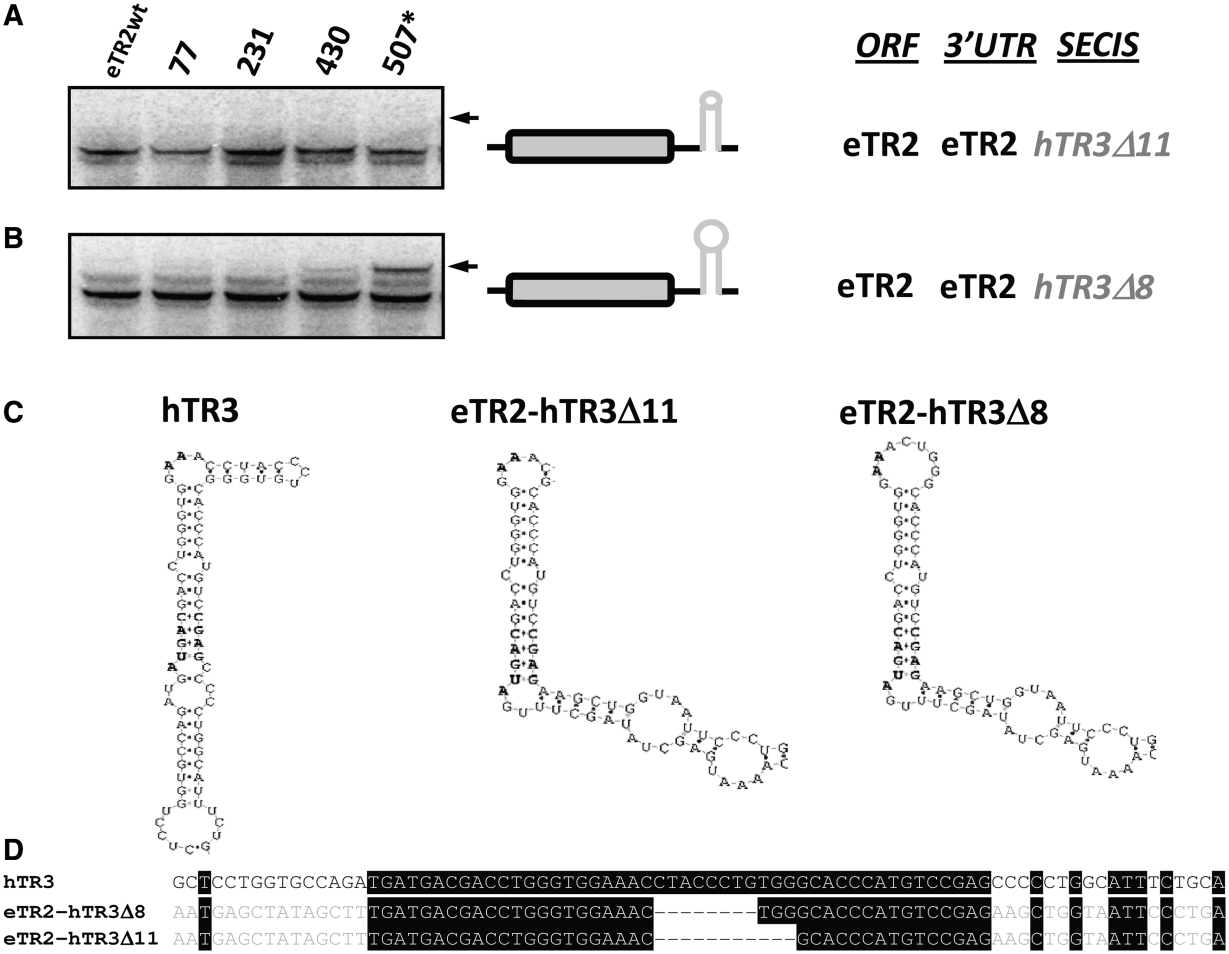
Role of the apical loop in hTR3 SECIS element in Sec insertion. (**A**) Sec insertion in eTR2 with the SECIS element from hTR3 with the 11 nt deletion in the apical loop. (**B**) Sec insertion in eTR2 with the SECIS element from hTR3 with the 8 nt deletion in the apical loop. (**C**) Natural and modified hTR3 SECIS elements used in the study. (**D**) Multiple sequence alignment of the natural and modified hTR3 SECIS elements. The experiment was carried out as described in Figure 2. Δ11 indicates deletion of 11 nt in the apical loop of the SECIS element. Δ8 indicates deletion of 8 nt in the apical loop of SECIS element.

We further analyzed the SECIS structure itself, examining the hTR3 SECIS element. Two mutant hTR3 SECIS elements were tested: *hTR3Δ11*, in which 11 nt were removed from the apical loop, and *hTR3Δ8*, in which 8 nt were deleted (Figure 6). The *hTR3Δ11* mutant was unable to support Sec insertion at any position owing to the extremely small apical loop, whereas the *hTR3Δ8* mutant made Sec insertion slightly less efficient (Figure 6A–D). A qPCR analysis showed no difference in mRNA levels for both sets of SECIS deletion constructs (Supplementary Figure S21).

### Structure of SECIS element influences position-dependent Sec insertion

SECIS elements are classified into two types based on the occurrence of a mini-stem within the apical loop (Figure 1A). However, belonging to a particular SECIS class does not necessarily characterize SECIS ‘strength’—the ability to efficiently insert Sec (10). As a rule, most Type II SECIS elements are predicted to be more thermodynamically stable than Type I structures. For example, the minimum free energy of *Euplotes* eTR2 SECIS element (Type I) is −19.10 kcal/mol, whereas for human hTR3 SECIS element (Type II), it is −30.70 kcal/mol. However, it is not clear how this relates to their ability to insert Sec. All *E. crassus* SECIS elements are of Type I structure and replacement of the eTR1 SECIS with that of *Toxoplasma gondii* Selenoprotein T (Type II structure) could relieve the restriction on insertion of Sec into natural positions (12). To further examine the eTR1 mRNA elements responsible for UGA codon position-dependent Sec insertion, we mutated a six-nucleotide sequence in the eTR1 ORF that showed complementarity with the eTR1 SECIS element (Supplementary Figure S22). This experiment tested a possibility that SECIS elements may exist in the form of alternative structures that are responsible for the observed effects. In addition, we deleted a long stable RNA structure found immediately downstream of the natural Sec UGA codon (Supplementary Figure S23). In this case, a possibility was examined that this structure delays progression of the ribosome, thereby promoting Sec insertion. However, these changes did not affect the coding function of UGA codons, i.e. Sec insertion was still restricted to canonical positions (Supplementary Figures S22 and S23). These findings support the notion that the SECIS element itself is responsible for such restriction. We also examined eSelW, a small 8 kDa selenoprotein with Sec located in the N-terminal region at position 9. This protein also has a second in-frame UGA codon located at position 85. Sec insertion into eSelW was tested using several mutant constructs with the EGFP fused at the N-terminus. Surprisingly, we found that Sec was inserted more efficiently at position 85 than at position 9 (Supplementary Figure S24A). Western blot analysis revealed a high level of truncated forms of the EGFP-eSelW fusion protein with the UGA at position 9 resembling the situation with the WT construct with the two UGA codons. Considering the fact that such a fusion protein may be unstable, we examined Sec insertion using a different tagged eSelW. Similarly to the EGFP fusion, Sec was inserted into eSelW with the N-terminal Myc-tag more efficiently at position 85. (Supplementary Figure S24B) These findings support the model of UGA codon-dependent and selenoprotein-specific Sec insertion controlled by the SECIS element structure, although this protein may also represent a more unique case of position-dependent Sec insertion.

## DISCUSSION

To test whether Sec insertion depends on the UGA codon position, we used mammalian cytosolic, mitochondrial and testes-specific thioredoxin reductases (hTR1, hTR3 and mTGR, respectively). Starting with natural TRs, we generated sets of mutant and chimeric constructs that helped us to examine various features of SECIS elements and selenoprotein 3′-UTRs. Expression of TRs from these constructs in HEK 293 cells, together with metabolic labeling of cells with ^75^Se, offered a simple, convenient and reproducible method to assay Sec insertion. We found that Sec insertion into hTR1 was strictly dependent on the location of the UGA codon. However, the UGA codon position in hTR3 had no influence on Sec insertion (i.e. Sec could be efficiently inserted into any place in the protein). Replacement of the 3′-UTR or SECIS element with those of *Euplotes* selenoproteins changed the UGA coding function in TR3, but did not affect it in TR1. For example, replacement of the 3′-UTR of hTR3 or its SECIS element with the corresponding regions of eTR2 restricted Sec insertion into the C-terminal part of the mammalian protein. In addition, replacement of the 3′-UTR or SECIS element of eTR2 with the corresponding sequences from hTR3 led to the insertion of Sec at previously restricted positions. Mammalian SECIS elements have been divided into two groups based on their secondary structure features, although the separation is not that strict, and the replacement of one SECIS type with another still supports Sec insertion (22–24). The relationship between SECIS type and UGA-position dependent Sec incorporation appears to be complex.

Before this work, it was assumed that Sec can be inserted into any position in mammalian selenoprotein sequences as well as in any position within the proteins with engineered Sec sites; i.e. having an in-frame UGA codon and a SECIS element in the 3′-UTR (at the right distance from the UGA codon) was thought to be sufficient for Sec insertion in mammalian cells. In contrast, we discovered that in human selenoproteins Sec insertion may be restricted, and that this residue is only incorporated into natural positions (sequences surrounding the natural positions also support Sec insertion). This surprising finding is consistent with the idea that a block in Sec insertion into unnatural positions was selected during evolution. One possibility is that this mechanism preserves selenoproteins within the domain of proteins regulated by nonsense-mediated decay. Indeed, if an additional UGA codon evolves in a selenoprotein, Sec insertion in this position would be detrimental, and the protein will likely be non-functional. Thus, removal of such mRNA through nonsense-mediated decay would benefit an organism.

A further twist in the positional dependence of Sec insertion is the finding that Sec insertion is limited to its natural position in a selenoprotein-specific manner, i.e. in some selenoproteins (TR1, TGR), Sec can only be inserted into the natural sites (and proximal sequences to these sites), whereas in TR3, this rule did not apply. What could be the mechanism for the position-dependent Sec insertion, or lack of it? It appears that the signal lies in the SECIS element and its specific structure, suggesting the role of interaction with SBP2. Indeed, several studies have shown that the SBP2-SECIS interaction is crucial for decoding UGA as Sec, and that the SECIS structure modulates this interaction and Sec insertion (10,15,25). Moreover, several other SECIS-interacting protein factors have been identified including nucleolin and the initiation factor 4a3 (26–28).

Questions remain regarding the role of Sec location in N-terminal versus C-terminal regions as well as the role of multiple SECIS elements. We observed that eSelW supported Sec insertion in both natural N-terminal and unnatural C-terminal sites, with the latter being more efficient. On the other hand, the use of the human SelP 3′-UTR, with its two SECIS elements, supported Sec inserted at any position, with the insertion into the N-terminal region being more efficient. It is an attractive possibility that the reason SelP has two SECIS elements is the need to insert Sec into two distant regions of the protein. Indeed, the first Sec is inserted into the N-terminal segment, whereas all other Sec residues are inserted into the C-terminal region. This possibility is consistent with the observation that the second SelP SECIS element is required for insertion of the N-terminal Sec, whereas the first SECIS element inserts C-terminal Sec residues (29). This observation, previously unclear, can be explained by the UGA codon position-dependent Sec insertion. Thus, it is not the matter of using two SECIS elements for insertion of multiple Sec residues, but the location of UGA codons in the coding sequence. This model suggests that two SECIS elements will be needed to insert two Sec residues into distant regions of the protein, whereas one SECIS element can support insertion of multiple Sec residues, provided they are located in the same region. One SECIS element may also be sufficient for insertion of multiple Sec residues if the protein lacks the ability for position-dependent Sec insertion. This is exactly the situation we observe from comparative selenoprotein analyses in thousands of completely sequenced genomes.

Finally, our findings shed new light on the use of artificial expression constructs, e.g. those in which UGA codon read-through allows synthesis of reporter proteins. Although such constructs allow comparison of efficiency of SECIS elements, they may not always reflect the natural situation and the natural SECIS element function, as many natural selenoproteins evolved to limit Sec insertion into much of their sequences. It would be important to reexamine SECIS element efficiency in light of the findings reported in our article.

## SUPPLEMENTARY DATA

Supplementary Data are available at NAR Online: Supplementary Table 1 and Supplementary Figures 1–24.

## FUNDING

National Institutes of Health (NIH) grants [GM065204 and GM061603 to V.N.G.] and the Intramural Research Program at the Center for Cancer Research, NCI, NIH (to D.L.H.). Funding for open access charge: NIH [GM065204 and GM061603].

*Conflict of interest statement*. None declared.

## Supplementary Material

Supplementary Data
