# Supplementary material for: UGA codon position-dependent incorporation of selenocysteine into mammalian selenoproteins
Source: Nucleic Acids Res. 2013 May 28;41(14):6952–9. doi: 10.1093/nar/gkt409 (PMC3737529; doi:10.1093/nar/gkt409)
Supplement: Supplementary Data [file supp_41_14_6952__index.html]

UGA codon position-dependent incorporation of selenocysteine into mammalian selenoproteins — UGA codon position-dependent incorporation of selenocysteine into mammalian selenoproteins — Supplementary Data 

# UGA codon position-dependent incorporation of selenocysteine into mammalian selenoproteins

## Supplementary Data

files

**Files in this Data Supplement:**

- Supplementary Data - pdf file
