# Supplementary material for: UGA codon position-dependent incorporation of selenocysteine into mammalian selenoproteins
Source: Nucleic Acids Res. 2013 May 28;41(14):6952–9. doi: 10.1093/nar/gkt409 (PMC3737529; doi:10.1093/nar/gkt409)
Supplement: Supplementary Data [file supp_gkt409_nar-00353-v-2013-File008.pdf]

Homo sapiens thioredoxin reductase 1 (TR1, TXNRD1), BC018122

AACGGCCCTGAAGATCTTCCCAAGTCCTATGACTATGACCTTATCATCATTGGAGGTGGCTCAGGAGGTCTGGCAGCT
GCTAAGGAGGCAGCCCAATATGGCAAGAAGGTGATGGTCCTGGACTTTGTCACTCCCACCCCTCTTGGAACTAGATGG
GGTCTCGGAGGAACATGTGTGAATGTGGGTTGCATACCTAAAAAACTGATGCATCAAGCAGCTTTGTTAGGACAAGCC
CTGCAAGACTCTCGAAATTATGGATGGAAAGTCGAGGAGACAGTTAAGCATGATTGGGACAGAATGATAGAAGCTGTA
CAGAATCACATTGGCTCTTTGAATTGGGGCTACCGAGTAGCTCTGCGGGAGAAAAAAGTCGTCTATGAGAATGCTTAT
GGGCAATTTATTGGTCCTCACAGGATTAAGGCAACAAATAATAAAGGCAAAGAAAAAATTTATTCAGCAGAGAGATTT
CTCATTGCCACTGGTGAAAGACCACGTTACTTGGGCATCCCTGGTGACAAAGAATACTGCATCAGCAGTGATGATCTT
TTCTCCTTGCCTTACTGCCCGGGTAAGACCCTGGTTGTTGGAGCATCCTATGTCGCTTTGGAGTGCGCTGGATTTCTT
GCTGGTATTGGTTTAGACGTCACTGTTATGGTTAGGTCCATTCTTCTTAGAGGATTTGACCAGGACATGGCCAACAAA
ATTGGTGAACACATGGAAGAACATGGCATCAAGTTTATAAGACAGTTCGTACCAATTAAAGTTGAACAAATTGAAGCA
GGGACACCAGGCCGACTCAGAGTAGTAGCTCAGTCCACCAATAGTGAGGAAATCATTGAAGGAGAATATAATACGGTG
ATGCTGGCAATAGGAAGAGATGCTTGCACAAGAAAAATTGGCTTAGAAACCGTAGGGGTGAAGATAAATGAAAAGACT
GGAAAAATACCTGTCACAGATGAAGAACAGACCAATGTGCCTTACATCTATGCCATTGGCGATATATTGGAGGATAAG
GTGGAGCTCACCCCAGTTGCAATCCAGGCAGGAAGATTGCTGGCTCAGAGGCTCTATGCAGGTTCCACTGTCAAGTGT
GACTATGAAAATGTTCCAACCACTGTATTTACTCCTTTGGAATATGGTGCTTGTGGCCTTTCTGAGGAGAAAGCTGTG
GAGAAGTTTGGGGAAGAAAATATTGAGGTTTACCATAGTTACTTTTGGCCATTGGAATGGACGATTCCGTCAAGAGAT
AACAACAAATGTTATGCAAAAATAATCTGTAATACTAAAGACAATGAACGTGTTGTGGGCTTTCACGTACTGGGTCCA
AATGCTGGAGAAGTTACACAAGGCTTTGCAGCTGCGCTCAAATGTGGACTGACCAAAAAGCAGCTGGACAGCACAATT
GGAATCCACCCTGTCTGTGCAGAGGTATTCACAACATTGTCTGTGACCAAGCGCTCTGGGGCAAGCATCCTCCAGGCT
GGCTGCTGAGGTTAA*GCCCCAGTGTGGATGCTGTTGCCAAGACTGCAAACCACTGGCTCGTTTCCGTGCCCAAATCCA*
AGGCGAAGTTTTCTAGAGGGTTCTTGGGCTCTTGGCACCTGCGTGTCCTGTGCTTACCACCGCCCAAGGCCCCCTTGG
ATCTCTTGGATAGGAGTTGGTGAATAGAAGGCAGGCAGCATCACACTGGGGTCACTGACAGACTTGAAGCTGACATTT
GGCAGGGCATCGAAGGGATGCATCCATGAAGTCACCAGTCTCAAGCCCATGTGGTAGGCGGTGATGGAACAACTGTCA
AATCAGTTTTAGCATGACCTTTCCTTGTGGATTTTCTTATTCTCGTTGTCAAGTTTTCTAGGGTTGAATTTTTTTCTT
TTTTCTCCATGGTGTTAATGATATTAGAGATGAAAAACGTTAGCAGTTGATTTTTGTCCAAAAGCAAGTCATGGCTAG
AGTATCCATGCAAGGTGTC

Figure S1. Nucleotide sequence of hTR1. The ORF is shown in black and 3'-UTR sequence used in the study is shown in grey and italic. The Sec-encoding UGA and the UAA stop codons are shown in red. The sequence corresponding to the core of the SECIS element is also shown in red.

Homo sapiens thioredoxin reductase 2 (TR3, TXNRD2), NM_006440

GGTCAGCGGGACTATGATCTCCTGGTGGTCGGCGGGGGATCTGGTGGCCTGGCTTGTGCCAAGGAGGCCGCCCAGCTG
GGAAGGAAGGTGGCCGTGGTGGACTACGTGGAACCTTCTCCCCAAGGCACCCGGTGGGGCCTCGGCGGCACCTGCGTC
AACGTGGGCTGCATCCCCAAGAAGCTGATGCACCAGGCGGCACTGCTGGGAGGCCTGATCCAAGATGCCCCCAACTAT
GGCTGGGAGGTGGCCCAGCCCGTGCCGCATGACTGGAGGAAGATGGCAGAAGCTGTTCAAAATCACGTGAAATCCTTG
AACTGGGGCCACCGTGTCCAGCTTCAGGACAGAAAAGTCAAGTACTTTAACATCAAAGCCAGCTTTGTTGACGAGCAC
ACGGTTTGCGGCGTTGCCAAAGGTGGGAAAGAGATTCTGCTGTCAGCCGATCACATCATCATTGCTACTGGAGGGCGG
CCGAGATACCCCACGCACATCGAAGGTGCCTTGGAATATGGAATCACAAGTGATGACATCTTCTGGCTGAAGGAATCC
CCTGGAAAAACGTTGGTGGTCGGGGCCAGCTATGTGGCCCTGGAGTGTGCTGGCTTCCTCACCGGGATTGGGCTGGAC
ACCACCATCATGATGCGCAGCATCCCCCTCCGCGGCTTCGACCAGCAAATGTCCTCCATGGTCATAGAGCACATGGCA
TCTCATGGCACCCGGTTCCTGAGGGGCTGTGCCCCCTCGCGGGTCAGGAGGCTCCCTGATGGCCAGCTGCAGGTCACC
TGGGAGGACAGCACCACCGGCAAGGAGGACACGGGCACCTTTGACACCGTCCTGTGGGCCATAGGTCGAGTCCCAGAC
ACCAGAAGTCTGAATTTGGAGAAGGCTGGGGTAGATACTAGCCCCGACACTCAGAAGATCCTGGTGGACTCCCGGGAA
GCCACCTCTGTGCCCCACATCTACGCCATTGGTGACGTGGTGGAGGGGCGGCCTGAGCTGACACCCATAGCGATCATG
GCCGGGAGGCTCCTGGTGCAGCGGCTCTTCGGCGGGTCCTCAGATCTGATGGACTACGACAATGTTCCCACGACCGTC
TTCACCCCGCTGGAGTATGGCTGTGTGGGGCTGTCCGAGGAGGAGGCAGTGGCTCGCCACGGGCAGGAGCATGTTGAG
GTCTATCACGCCCATTATAAACCACTGGAGTTCACGGTGGCTGGACGAGATGCATCCCAGTGTTATGTAAAGATGGTG
TGCCTGAGGGAGCCCCCACAGCTGGTGCTGGGCCTGCATTTCCTTGGCCCCAACGCAGGCGAAGTTACTCAAGGATTT
GCTCTGGGGATCAAGTGTGGGGCTTCCTATGCGCAGGTGATGCGGACCGTGGGTATCCATCCCACATGCTCTGAGGAG
GTAGTCAAGCTGCGCATCTCCAAGCGCTCAGGCCTGGACCCCACGGTGACAGGCTGCTGAGGGTAA*GCGCCATCCCTG*
CAGGCCAGGGCACACGGTGCGCCCGCCGCCAGCTCCTCGGAGGCCAGACCCAGGATGGCTGCAGGCCAGGTTTGGGGG
GCCTCAACCCTCTCCTGGAGCGCCTGTGAGATGGTCAGCGTGGAGCGCAAGTGCTGGACAGGTGGCCCGTGTGCCCCA
CAGGGATGGCTCAGGGGACTGTCCACCTCACCCCTGCACCTCTCAGCCTCTGCCGCCGGGCACCCCCCCCCAGGCTCC
TGGTGCCAGATGATGACGACCTGGGTGGAAACCTACCCTGTGGGCACCCATGTCCGAGCCCCCTGGCATTTCTGCAAT
GCAAATAAAGAGGGTACTTTTTCTGAAGTGTG

Figure S2. Nucleotide sequence of hTR3. The ORF is shown in black and the 3'-UTR sequence used in the study is shown in grey and italic. The Sec-encoding UGA and the UAA stop codons are shown in red. The sequence corresponding to the core of the SECIS element is also shown in red.

Mus musculus thioredoxin reductase 3 (TGR, TXNRD3), NM_153162

GAACAAAAACTTATTTCTGAAGAAGATCTGTCGTCGCCACCCGGCCGCCGCGCCCGCCTGGCCTCCCCTGGGACCAGCCGC
CCGTCTTCTGAGGCCCGCGAGGAGCTGCGGCGCCGCCTGCGGGACCTCATCGAGGGCAACAGGGTGATGATCTTCAGC
AAGAGTTACTGTCCACACAGCACGCGGGTTAAGGAACTCTTTTCGTCCTTGGGAGTGGTCTATAACATCCTGGAACTT
GATCAAGTTGATGACGGGGCCAGTGTTCAGGAAGTGCTGACAGAAATCAGTAACCAGAAAACGGTGCCCAATATTTTT
GTGAATAAAGTGCACGTGGGTGGATGTGACCGAACTTTCCAGGCACATCAGAATGGTTTACTGCAGAAGCTCCTCCAA
GATGACTCGGCTCATGATTACGACCTCATCATCATCGGCGGGGGTTCTGGCGGCCTCTCTTGTGCCAAGGAAGCTGCC
AACTTGGGAAAGAAGGTCATGGTGCTAGACTTTGTGGTCCCATCGCCTCAGGGCACGACCTGGGGCCTTGGCGGCACC
TGTGTGAACGTAGGCTGTATTCCAAAGAAGCTGATGCATCAGGCAGCCCTCCTGGGGCATGCTTTGCAAGATGCCAAG
AAATATGGCTGGGAGTATAACCAGCAGGTGAAGCACAACTGGGAGGCCATGACAGAAGCTATCCAGAGCCACATTGGC
TCCTTGAACTGGGGCTACAGGGTAACCCTTCGGGAGAAAGGCGTGACCTATGTCAACTCCTTCGGGGAGTTTGTGGAC
CTGCATAAAATAAAGGCAACCAATAAGAAAGGGCAGGAAACGTTTTACACGGCTTCGAAGTTTGTCATAGCAACAGGT
GAAAGGCCTCGGTACTTGGGAATCCAGGGAGATAAAGAGTACTGTATTACGAGTGACGACCTGTTCTCTCTGCCATAC
TGCCCGGGATGTACATTAGTCGTAGGCGCTTCTTATGTTGGTCTGGAGTGTGCGGGCTTTTTGGCTGGTTTGGGGTTA
GATGTGACAGTCATGGTACGCTCCGTCCTTCTTCGTGGCTTTGATCAAGAAATGGCAGAGAAAGTGGGATCCTACCTG
GAACAACAAGGTGTCAAGTTCCAAAGGAAATTCACCCCCATTTTGGTTCAACAGTTGGAGAAAGGTTTACCAGGAAAA
TTGAAAGTCGTGGCTAAGTCCACCGAAGGACCGGAAACAGTAGAAGGGATATACAACACGGTTTTGTTAGCAATTGGT
CGTGACTCCTGTACAAGGAAAATAGGGCTGGAGAAGATCGGGGTCAAAATCAATGAGAAGAATGGCAAAATACCAGTA
AACGATGTGGAGCAGACCAACGTGCCTCATGTTTATGCTATTGGGGACATACTGGACGGCAAACCAGAGCTCACCCCC
GTTGCCATACAGGCAGGCAAGCTGCTAGCTCGAAGACTCTTTGGGGTCTCTTTAGAAAAGTGTGATTATATTAACATC
CCAACAACGGTGTTCACACCTCTGGAATATGGCTGTTGTGGACTGTCGGAAGAGAAAGCCATCGAAATGTATAAAAAA
GAGAATCTGGAAGTGTATCACACCTTGTTTTGGCCTCTCGAGTGGACAGTTGCTGGCAGAGACAACAACACCTGTTAT
GCAAAGATAATCTGCAACAAATTCGACAACGAACGTGTGGTGGGATTTCACCTTCTGGGGCCAAATGCTGGTGAAATC
ACGCAGGGATTTGCAGCTGCAATGAAATGTGGGCTCACGAAGCAGCTACTGGATGATACCATTGGAATCCACCCCACC
TGTGGTGAGGTATTCACAACATTGGAAATCACAAAGTCCTCAGGGCTGGACATTACTCAGAAAGGCTGCTGAGGCTAG
CCCTGCTGCTGTTGGGTTTCCCTGTCACACTCTCACTCTGTGCCCATGACATCTGCTTGGGCACTGGGGACCAGTGCC
GGACTTTCTCCTGGCATAGAGACGAGAAGGCGGCAGAGGACCAGCAGCAGCAGCAGCATCTGGAGTCTCAGCTGACGG
CATGCAGCAGCCAGGCTGCTTCCTTGACACCTTGGCTCGGAACCTGCAGAGGTGAGCCAAGGCCGACTTCTGCACGTC
AGCCTCGACTTCACCCCACGAGGCCTTAGATGGCACAGTGAGCCCACCTCCCTTTCCCAGTTGCTAGTGCTTTGACCC
CTCACTTTGTTTCCATGAAGAGATTATCTTTCAGTTGTGGACTTGACTGTAAAGAGGCTTGTTGCCCAGAGTGTAGCA
CTTATGGATCTGTGGTGTTTGGGGAGCCAGGTTAGAGC

Figure S3. Nucleotide sequence of mTGR. The ORF is shown in black and the 3'-UTR sequence used in the study is shown in grey and italic. The Sec-encoding UGA and the UAG stop codons are shown in red. The sequence corresponding to the core of the SECIS element is also shown in red. The sequence corresponding to the N-terminal myc-tag is shown in bold and is underlined.

Euplotes crassus thioredoxin reductase 1 (eTR1)

GACTATTCAGACACTCCACAAGAAGAATCCACTCATAGTTATGACTATGATCTCTTTGTAATCGGAGGTGGTTCTGGA
GGGCTTGCTTGTGCCAAGGCTGCTCAAGAAGCAGGAGCTAAAGTAGCAGTAGCTGATTTTGTAAAGCCAACTCCAAAG
GGAACAAAGTGGAAAGTAGGAGGAACATGAGTGAATGTTGGTTGAATCCCCAAAAAGCTGATGCACTACTCCGCATTG
TTAGGAAATTCTTATCACGACCAAGTTGAGAGCGGATGGGAGCATGAGAAACCTTCTCATGACTGGGGTAAAATGATT
ACCAATGTCAATAACCATATAAGAGGTATCAATTTTGGATACAAAGCAGATATGAGAAAGAGAGGTATAAAGTTTCAT
GAAAAGTTTGCATCCTTTGTCGATCCTCATACCGTACAACTAGTTGATAAGAAGGGCAAGACCGAAATGATTACTTCT
AATTATTTCGTAATTGCTACTGGAGGCAGACCTCTCTATCCTGATATTCCAGGAGCCAAAGAGCATGCAATTACTAGT
GATGATATTTTCTGGATGAAAGACAACCCTGGTAAAACCCTTGTGGTAGGTGCTTCCTATGTCGCGTTGGAATGAGCT
GGATTTTTACATCATTTTGGAAACGAAGTTTCAGTTTGTGTCCGATCAATCTTTTTGAGAGGTTTCGATCAAGATATG
GCGCAAAAGATTGCTAAAGACATGGAACTCAGCGGGATTAATTTCATTAGAGACTCTGTGCCTACCAAAATTGAGAAA
GACGAAGAGACTGGCAAGCTCACCTGATTTTTAACAGTAGGAGGCGAAGAAACTACCGTTGAAGTAGATACTGTTCTT
TTTGCAATTGGTAGATATGCTGTGACAGCTGATTTAAATCTAGGTAATGCTGGACTCATTGCTGAAAAGAATGGAAAA
TTCATTACTGACAAATACCAGAAAACTAATGTTGACAATATCTATGCTATAGGGGATGTGCTTCATGGAAAATTGGAA
CTTACTCCAACTGCAATTCAAGCGGGAAGACTATTGGCTGATAGACTATTTGCTGGAGGAACTACTACAATGGATTTT
TATGATGTTCCTACTACTATCTTTACTCCTCTTGAGTATGGATGAGTAGGTTACTCAGAGGAAGATGCTAGGGAGGAA
TATGGTGACTTCATCAAAGTTTACCATACTTATTTCCAGCCATTAGAATGGAACTTTGCAAAATCAATCTATAAGGAG
AGGAATTGATACGTAAAAATTATAATCAACACTGCAGATAATGACAGAGTGATCGGGTTCCATATTCTCTGTCCTAAT
GCTGGGGAGATAACACAAGGAATTGCTATTGCCATCAAAGTAGGAGTCACAAAGCCTCAGTTAGATAACTGTGTTGGA
ATTCATCCTACAATTGCTGAAGAAATGACTAATCTACATATTGATAAAGCTGATAATCCAGATCCAATTAAATCGGAT
TGCTGATCTTAA*AGAGTCTGTGCATATTAGGAGTTGCTAAAATTTAGCCAACTACTAATAACTGCATGGAAAAATAT*
AGTCGGTAAAGGAGACAAATCAGTAAGCAATGTTTCAAGACAATATGCTTGGTCTTTAGAAGCATTAGCTTTAATGAT
ACTCTCTTTCTTCAAATACATAAGAAAGCGAGTTGAACAGCTGAGGACTCTCTGAGTAGTAATGGATCCACCG

Figure S4. Nucleotide sequence of eTR1. The ORF is shown in black and the 3’-UTR sequence used in the study is shown in grey and italic. The Sec-encoding UGA and the UAA stop codons are shown in red. The sequence corresponding to the core of the SECIS element is also shown in red.

Euplotes crassus thioredoxin reductase 2 (eTR2)

GCTGACTCTGAAATGAAGAGTAATGAGTCAGAAGAATATGAAGAGACTAAGCGTCGTTATGATTATGATCTCTTCGTC
ATTGGTGGAGGATCAGGAGGACTTGCTTGTGCTAAAGCAGCTCAAGAGTGTGGAGCAAAGGTAGCTGTAGCTGACTTT
GTCAAGCCCTCTCCTCATGGATATGGAGTTGTGACTTGGGGAGTTGGTGGAACTTGTGTCAATGTGGGATGAATACCA
AAGAAATTATTACATTATTCAGCAAATTTGGGAGAAGCTTATGTTGATAGAGCTAGTAGTGGATGGGACCATGAGAAG
CCAAAACACGATTGGGGTAAAATGATTTCTAATATTAATAATCATATTCGAGCTATTAATTTTAGCATCAAAACTGAT
TTGAGGAAGAGAGGAATAAAATTTTATGAAAAATTAGCTTCTTTTGCTGATCCACATACTATTCAACTTTTAAACAAG
AAAGGCAAGACAGAATTAGTGACAGCAAATCATATTGTTATTGCAACTGGGGGAAGGCCTCTCTACCCTGATATCCCT
GGAGCAAAGGAGTATGGTATTACAAGCGATGACATTTTCTGGCTGAAGAAAAATCCAGGTAAAACCTTGGTCATTGGC
GCATCTTATATTGCACTTGAATGAGCTGGATTTTTACATAGTTTTGGTAACGATGTTTCTGTGTGAGTAAGATCGGTC
TTTTTGCGGGGCTTTGATCAGGATATGGCTAATATGCTTGCCAAGGATATGGAAGAACATGGTGGAGTCAAATTCATT
AAAAATTCAATACCTACCAAAATCGAAAAAGATGAAGAAACAGGAAAGCTCATATGATATCTCACCTCTAGAGAAGAG
GAAATTACTATAGAAGTTGACACAGTTTTGTTTGCAATTGGTAGATATGCTGTTACAAAAGATCTAAACCTTGAAAAT
GCGGGTCTCAAAGTAGAATCAAACGGTAAATTCATTACAGATGAGTTTCAACAAACTAATGTGGAGAATATCTATGCT
ATCGGAGATGTGATTCATGGGAAATTAGAACTAACACCCACTGCAATTCAAACAGGTAAACTACTTGCAAGAAGATTG
TATGCTGGTGAAACCACAACTATGGACTTTTGTGATATTCCAACTACAATCTTCACTCCTTTAGAGTATGGATGAGTT
GGATACTCAGAAGAAGAAGCTAAGGAAAAATATGGAGACGCCATTAAGGTATATCATACTTACTTCAAGCCATTAGAG
TGGAACTATGCAAAATCAATTTATAAATATCGAAATTGATATGTTAAAGTAATTATAAACACTACAGAGAATGATCGG
GTAATTGGCTATCATTTATTGGCTCCAAATGCAGGAGAAATTACTCAAGGAATTGCAATTGCCATTAAGATTGGCCTT
ACTAAACACAAGTTAGATAACTGTGTTGGAATCCATCCAACTGTTGCAGAAGAAGTAACGGATCTCAAGATTGATAAA
GCAATCAATCCTGATCCAGTCAAGACAGATTGTTGATCTTAG*AGCATCTGTACATATTAGTCATTGCTTTTACAAAGG*
CAATCACTAATAGCAGTACGGAATAATTAATCTGAAAAGGGGAGGACCCAGTAAGCTATAGATCATACTCAAAATGAG
CTATAGCTTTTATGATACTTCTTTCCTTCAAAAATATAAAGGAAACAAGTTGAAAAGCTGGTAATTCCCTGAGCAGCT
TCAGCAATAATTG

Figure S5. Nucleotide sequence of hTR3. The ORF is shown in black and the 3'-UTR sequence used in the study is shown in grey and italic. The Sec-encoding UGA and the UAG stop codons are shown in red. The sequence corresponding to the core of the SECIS element is also shown in red.

hTR1-eTR1ORF+3’UTR

AACGGCCCTGAAGATCTTCCCAAGTCCTATGACTATGACCTTATCATCATTGGAGGTGGCTCAGGAGGTCTGGCAGCT
GCTAAGGAGGCAGCCCAATATGGCAAGAAGGTGATGGTCCTGGACTTTGTCACTCCCACCCCTCTTGGAACTAGATGG
GGTCTCGGAGGAACATGTGTGAATGTGGGTTGCATACCTAAAAAACTGATGCATCAAGCAGCTTTGTTAGGACAAGCC
CTGCAAGACTCTCGAAATTATGGATGGAAAGTCGAGGAGACAGTTAAGCATGATTGGGACAGAATGATAGAAGCTGTA
CAGAATCACATTGGCTCTTTGAATTGGGGCTACCGAGTAGCTCTGCGGGAGAAAAAAGTCGTCTATGAGAATGCTTAT
GGGCAATTTATTGGTCCTCACAGGATTAAGGCAACAAATAATAAAGGCAAAGAAAAAATTTATTCAGCAGAGAGATTT
CTCATTGCCACTGGTGAAAGACCACGTTACTTGGGCATCCCTGGTGACAAAGAATACTGCATCAGCAGTGATGATCTT
TTCTCCTTGCCTTACTGCCCGGGTAAGACCCTGGTTGTTGGAGCATCCTATGTCGCTTTGGAGTGCGCTGGATTTCTT
GCTGGTATTGGTTTAGACGTCACTGTTATGGTTAGGTCCATTCTTCTTAGAGGATTTGACCAGGACATGGCCAACAAA
ATTGGTGAACACATGGAAGAACATGGCATCAAGTTTATAAGACAGTTCGTACCAATTAAAGTTGAACAAATTGAAGCA
GGGACACCAGGCCGACTCAGAGTAGTAGCTCAGTCCACCAATAGTGAGGAAATCATTGAAGGAGAATATAATACGGTG
ATGCTGGCAATAGGAAGAGATGCTTGCACAAGAAAAATTGGCTTAGAAACCGTAGGGGTGAAGATAAATGAAAAGACT
GGAAAAATACCTGTCACAGATGAAGAACAGACCAATGTGCCTTACATCTATGCCATTGGCGATATATTGGAGGATAAG
GTGGAGCTCACCCCAGTTGCAATCCAGGCAGGAAGATTGCTGGCTCAGAGGCTCTATGCAGGTTCCACTGTCAAGTGT
GACTATGAAAATGTTCCAACCACTGTATTTACTCCTTTGGAATATGGTGCTTGTGGCCTTTCTGAGGAGAAAGCTGTG
GAGAAGTTTGGGGAAGAAAATATTGAGGTTTACCATAGTTACTTTTGGCCATTGGAATGGACGATTCCGTCAAGAGAT
AACAACAAATGTTATGCAAAAATAATCTGTAATACTAAAGACAATGAACGTGTTGTGGGCTTTCACGTACTGGGTCCA
AATGCTGGAGAAGTTACACAAGGCTTTGCAGCTGCGCTCAAATGTGGACTGACCAAAAAGCAGCT**G**TCGACCTGTGTT
GGAATTCATCCTACAATTGCTGAAGAAATGACTAATCTACATATTGATAAAGCTGATAATCCAGATCCAATTAAATCG
GATTGCTGATCTTAA*AGAGTCTGTGCATATTAGGAGTTGCTAAAATTTAGCCAACTACTAATAACTGCATGGGAAAAA*
TATAGTCGGTAAAGGAGACAAATCAGTAAGCAATGTTTCAAGACAATATGCTTGGTCTTTAGAAGCATTAGCTTTAAT
GATACTCTCTTTCTTCAAATACATAAGAAAGCGAGTTGAACAGCTGAGGACTCTCTGAGTAGTAAT

Figure S6. Nucleotide sequence of the chimeric hTR1-eTR1 construct. The ORF is shown in black and the 3’-UTR sequence used in the study is shown in grey and italic. The Sec-encoding UGA and the UAA stop codons are shown in red. The sequence corresponding to the eTR1 ORF is underlined. The sequence corresponding to the core of the SECIS element is shown in red.

hTR1-eTR1Secis

AACGGCCCTGAAGATCTTCCCAAGTCCTATGACTATGACCTTATCATCATTGGAGGTGGCTCAGGAGGTCTGGCAGCT
GCTAAGGAGGCAGCCCAATATGGCAAGAAGGTGATGGTCCTGGACTTTGTCACTCCCACCCCTCTTGGAACTAGATGG
GGTCTCGGAGGAACATGTGTGAATGTGGGTTGCATACCTAAAAAACTGATGCATCAAGCAGCTTTGTTAGGACAAGCC
CTGCAAGACTCTCGAAATTATGGATGGAAAGTCGAGGAGACAGTTAAGCATGATTGGGACAGAATGATAGAAGCTGTA
CAGAATCACATTGGCTCTTTGAATTGGGGCTACCGAGTAGCTCTGCGGGAGAAAAAAGTCGTCTATGAGAATGCTTAT
GGGCAATTTATTGGTCCTCACAGGATTAAGGCAACAAATAATAAAGGCAAAGAAAAAATTTATTCAGCAGAGAGATTT
CTCATTGCCACTGGTGAAAGACCACGTTACTTGGGCATCCCTGGTGACAAAGAATACTGCATCAGCAGTGATGATCTT
TTCTCCTTGCCTTACTGCCCGGGTAAGACCCTGGTTGTTGGAGCATCCTATGTCGCTTTGGAGTGCGCTGGATTTCTT
GCTGGTATTGGTTTAGACGTCACTGTTATGGTTAGGTCCATTCTTCTTAGAGGATTTGACCAGGACATGGCCAACAAA
ATTGGTGAACACATGGAAGAACATGGCATCAAGTTTATAAGACAGTTCGTACCAATTAAAGTTGAACAAATTGAAGCA
GGGACACCAGGCCGACTCAGAGTAGTAGCTCAGTCCACCAATAGTGAGGAAATCATTGAAGGAGAATATAATACGGTG
ATGCTGGCAATAGGAAGAGATGCTTGCACAAGAAAAATTGGCTTAGAAACCGTAGGGGTGAAGATAAATGAAAAGACT
GGAAAAATACCTGTCACAGATGAAGAACAGACCAATGTGCCTTACATCTATGCCATTGGCGATATATTGGAGGATAAG
GTGGAGCTCACCCCAGTTGCAATCCAGGCAGGAAGATTGCTGGCTCAGAGGCTCTATGCAGGTTCCACTGTCAAGTGT
GACTATGAAAATGTTCCAACCACTGTATTTACTCCTTTGGAATATGGTGCTTGTGGCCTTTCTGAGGAGAAAGCTGTG
GAGAAGTTTGGGGAAGAAAATATTGAGGTTTACCATAGTTACTTTTGGCCATTGGAATGGACGATTCCGTCAAGAGAT
AACAACAAATGTTATGCAAAAATAATCTGTAATACTAAAGACAATGAACGTGTTGTGGGCTTTCACGTACTGCGTCCA
AATGCTGGAGAAGTTACACAAGGCTTTGCAGCTGCGCTCAAATGTGGACTGACCAAAAAGCAGCTGGACAGCACAATT
GGAATCCACCCTGTCTGTGCAGAGGTATTCACAACATTGTCTGTGACCAAGCGCTCTGGGGCAAGCATCCTCCAGGCT
GGCTGCTGAGGTTAA*GCCCCAGTGTGGATGCTGTTGCCAAGACTGCAAACCACTGGCTCGTTTCCGTGCCCAAATCCA*
AGGCGAAGTTTTCTAGAGGGTTCTTGGGCTCTTGGCACCTGCGTGTCCTGTGCTTACCACCGCCCAAGGCCCCCTTGG
ATCTCTTGGATAGGAGTTGGTGAATAGAAGGCAGGCAGCATCACACTGGGGTCACTGACAGACTTGAAGCTGACATTT
*GGCAGGGCATCGAAGGATGCATC**C**ATGATACTCTCTTTCTTCAAATACATAAGAAAGCGAGTTGAACAACTGTCAAA*
TCAGTTTTAGCATGACCTTTCCTTGTGGATTTTCTTATTCTCGTTGTCAAGTTTTCTAGGGTTGAATTTTTTTCTTTT
TTCTCCATGGTGTTAATGATATTAGAGATGAAAAACGTTAGCAGTTGATTTTTGTCCAAAAGCAAGTCATGGCTAGAG
TATCCATGCAAGGTGTC

Figure S7. Nucleotide sequence of the chimeric hTR1-eTR1 SECIS construct. The ORF is shown in black and the 3’-UTR sequence is shown in grey and italic. The Sec-encoding UGA and the UAA stop codons are shown in red. The sequence corresponding to the core of the SECIS element is also shown in red.

hTR3-eTR2ORF+3′UTR

GGTCAGCGGGACTATGATCTCCTGGTGGTCGGCGGGGGATCTGGTGGCCTGGCTTGTGCCAAGGAGGCCGCCCAGCTG
GGAAGGAAGGTGGCCGTGGTGGACTACGTGGAACCTTCTCCCCAAGGCACCCGGTGGGGCCTCGGCGGCACCTGCGTC
AACGTGGGCTGCATCCCCAAGAAGCTGATGCACCAGGCGGCACTGCTGGGAGGCCTGATCCAAGATGCCCCCAACTAT
GGCTGGGAGGTGGCCCAGCCCGTGCCGCATGACTGGAGGAAGATGGCAGAAGCTGTTCAAAATCACGTGAAATCCTTG
AACTGGGGCCACCGTGTCCAGCTTCAGGACAGAAAAGTCAAGTACTTTAACATCAAAGCCAGCTTTGTTGACGAGCAC
ACGGTTTGCGGCGTTGCCAAAGGTGGGAAAGAGATTCTGCTGTCAGCCGATCACATCATCATTGCTACTGGAGGGCGG
CCGAGATACCCCACGCACATCGAAGGTGCCTTGGAATATGGAATCACAAGTGATGACATCTTCTGGCTGAAGGAATCC
CCTGGAAAAACGTTGGTGGTCGGGGCCAGCTATGTGGCCCTGGAGTGTGCTGGCTTCCTCACCGGGATTGGGCTGGAC
ACCACCATCATGATGCGCAGCATCCCCCTCCGCGGCTTCGACCAGCAAATGTCCTCCATGGTCATAGAGCACATGGCA
TCTCATGGCACCCGGTTCCTGAGGGGCTGTGCCCCCTCGCGGGTCAGGAGGCTCCCTGATGGCCAGCTGCAGGTCACC
TGGGAGGACAGCACCACCGGCAAGGAGGACACGGGCACCTTTGACACCGTCCTGTGGGCCATAGGTCGAGTCCCAGAC
ACCAGAAGTCTGAATTTGGAGAAGGCTGGGGTAGATACTAGCCCCGACACTCAGAAGATCCTGGTGGACTCCCGGGAA
GCCACCTCTGTGCCCCACATCTACGCCATTGGTGACGTGGTGGAGGGGCGGCCTGAGCTGACACCCATAGCGATCATG
GCCGGGAGGCTCCTGGTGCAGCGGCTCTTCGGCGGGTCCTCAGATCTGATGGACTACGACAATGTTCCCACGACCGTC
TTCACCCCGCTGGAGTATGGCTGTGTGGGGCTGTCCGAGGAGGAGGCAGTGGCTCGCCACGGGCAGGAGCATGTTGAG
GTCTATCACGCCCATTATAAACCACTGGAGTTCACGGTGGCTGGACGAGATGCATCCCAGTGTTATGTAAAGATGGTG
TGCCTGAGGGAGCCCCCACAGCTGGTGCTGGGCCTGCATTTCCTTGGCCCCAACGCAGGCGAAGTTACTCAAGGATTT
GCTCTGGGGATCAAGTGTGGGGCTTCCTATGCGCAGGTGATGCGGACCGTGGGTATCCATCCCACATGCTCTGTCGAC
GAAGAAGTAACGGATCTCAAGATTGATAAAGCAATCAATCCTGATCCAGTCAAGACAGATTGTTGATCTTAG*AGCATC*
TGTACATATTAGTCATTGCTTTTACAAAGGCAATCACTAATAGCAGTACGGAATAATTAATCTGAAAAGGGGAGGACC
CAGTAAGCTATAGATCATACTCAAAATGAGCTATAGCTTTTATGATACTTCTTTCCTTCAAAAATATAAAGGAAACAA
GTTGAAAAGCTGGTAATTCCCTGAGCAGCTTCAGCAATAATTG

Figure S8. Nucleotide sequence of the chimeric hTR3-eTR2 construct. The ORF is shown in black and the 3’-UTR sequence is shown in grey and italic. The Sec-encoding UGA and the UAG stop codons are shown in red. The sequence corresponding to the eTR2 ORF is underlined. The sequence corresponding to the core of the SECIS element is shown in red.

hTR3-eTR2Secis

GGTCAGCGGGACTATGATCTCCTGGTGGTCGGCGGGGGATCTGGTGGCCTGGCTTGTGCCAAGGAGGCCGCCCAGCTG
GGAAGGAAGGTGGCCGTGGTGGACTACGTGGAACCTTCTCCCCAAGGCACCCGGTGGGGCCTCGGCGGCACCTGCGTC
AACGTGGGCTGCATCCCCAAGAAGCTGATGCACCAGGCGGCACTGCTGGGAGGCCTGATCCAAGATGCCCCCAACTAT
GGCTGGGAGGTGGCCCAGCCCGTGCCGCATGACTGGAGGAAGATGGCAGAAGCTGTTCAAAATCACGTGAAATCCTTG
AACTGGGGCCACCGTGTCCAGCTTCAGGACAGAAAAGTCAAGTACTTTAACATCAAAGCCAGCTTTGTTGACGAGCAC
ACGGTTTGCGGCGTTGCCAAAGGTGGGAAAGAGATTCTGCTGTCAGCCGATCACATCATCATTGCTACTGGAGGGCGG
CCGAGATACCCCACGCACATCGAAGGTGCCTTGGAATATGGAATCACAAGTGATGACATCTTCTGGCTGAAGGAATCC
CCTGGAAAAACGTTGGTGGTCGGGGCCAGCTATGTGGCCCTGGAGTGTGCTGGCTTCCTCACCGGGATTGGGCTGGAC
ACCACCATCATGATGCGCAGCATCCCCCTCCGCGGCTTCGACCAGCAAATGTCCTCCATGGTCATAGAGCACATGGCA
TCTCATGGCACCCGGTTCCTGAGGGGCTGTGCCCCCTCGCGGGTCAGGAGGCTCCCTGATGGCCAGCTGCAGGTCACC
TGGGAGGACAGCACCACCGGCAAGGAGGACACGGGCACCTTTGACACCGTCCTGTGGGCCATAGGTCGAGTCCCAGAC
ACCAGAAGTCTGAATTTGGAGAAGGCTGGGGTAGATACTAGCCCCGACACTCAGAAGATCCTGGTGGACTCCCGGGAA
GCCACCTCTGTGCCCCACATCTACGCCATTGGTGACGTGGTGGAGGGGCGGCCTGAGCTGACACCCATAGCGATCATG
GCCGGGAGGCTCCTGGTGCAGCGGCTCTTCGGCGGGTCCTCAGATCTGATGGACTACGACAATGTTCCCACGACCGTC
TTCACCCCGCTGGAGTATGGCTGTGTGGGGCTGTCCGAGGAGGAGGCAGTGGCTCGCCACGGGCAGGAGCATGTTGAG
GTCTATCACGCCCATTATAAACCACTGGAGTTCACGGTGGCTGGACGAGATGCATCCCAGTGTTATGTAAAGATGGTG
TGCCTGAGGGAGCCCCCACAGCTGGTGCTGGGCCTGCATTTCCTTGGCCCCAACGCAGGCGAAGTTACTCAAGGATTT
GCTCTGGGGATCAAGTGTGGGGCTTCCTATGCGCAGGTGATGCGGACCGTGGGTATCCATCCCACATGCTCTGAGGAG
GTAGTCAAGCTGCGCATCTCCAAGCGCTCAGGCCTGGACCCCACGGTGACAGGCTGCTGAGGGTAA*GCGCCATCCCTG*
CAGGCCAGGGCACACGGTGCGCCCGCCGCCAGCTCCTCGGAGGCCAGACCCAGGATGGCTGCAGGCCAGGTTTGGGGG
GCCTCAACCCTCTCCTGGAGCGCCTGTGAGATGGTCAGCGTGGAGCGCAAGTGCTGGACAGGTGGCCCGTGTGCCCCA
CAGGGATGGCTCAGGGGACTGTCCACCTCACCCCTGCACCTCTCAGCCTCTGCCGCCGGGCACCCCCCCCAGGCTCC
TGGTGCCAGATGATGATACTTCTTTCCTTCAAAAATATAAAGGAAACAAGTTGAACCCCCTGGCATTTCTGCAATGCA
AATAAAGAGGGTACTTTTTCTGAAGTGTG

Figure S9. Nucleotide sequence of the chimeric hTR3-eTR2 SECIS construct. The ORF is shown in black and the 3’-UTR sequence is shown in grey and italic. The Sec-encoding UGA and the UAA stop codons are shown in red. The sequence corresponding to the core of the SECIS element is also shown in red.

eTR1-hTR1Secis

GACTATTCAGACACTCCACAAGAAGAATCCACTCATAGTTATGACTATGATCTCTTTGTAATCGGAGGTGGTTCTGGA
GGGCTTGCTTGTGCCAAGGCTGCTCAAGAAGCAGGAGCTAAAGTAGCAGTAGCTGATTTTGTAAAGCCAACTCCAAAG
GGAACAAAGTGGAAAGTAGGAGGAACATGAGTGAATGTTGGTTGAATCCCCAAAAAGCTGATGCACTACTCCGCATTG
TTAGGAAATTCTTATCACGACCAAGTTGAGAGCGGATGGGAGCATGAGAAACCTTCTCATGACTGGGGTAAAATGATT
ACCAATGTCAATAACCATATAAGAGGTATCAATTTTGGATACAAAGCAGATATGAGAAAGAGAGGTATAAAGTTTCAT
GAAAAGTTTGCATCCTTTGTCGATCCTCATACCGTACAACTAGTTGATAAGAAGGGCAAGACCGAAATGATTACTTCT
AATTATTTCGTAATTGCTACTGGAGGCAGACCTCTCTATCCTGATATTCCAGGAGCCAAAGAGCATGCAATTACTAGT
GATGATATTTTCTGGATGAAAGACAACCCTGGTAAAACCCTTGTGGTAGGTGCTTCCTATGTCGCGTTGGAATGAGCT
GGATTTTTACATCATTTTGGAAACGAAGTTTCAGTTTGTGTCCGATCAATCTTTTTGAGAGGTTTCGATCAAGATATG
GCGCAAAAGATTGCTAAAGACATGGAACTCAGCGGGATTAATTTCATTAGAGACTCTGTGCCTACCAAAATTGAGAAA
GACGAAGAGACTGGCAAGCTCACCTGATTTTTAACAGTAGGAGGCGAAGAAACTACCGTTGAAGTAGATACTGTTCTT
TTTGCAATTGGTAGATATGCTGTGACAGCTGATTTAAATCTAGGTAATGCTGGACTCATTGCTGAAAAGAATGGAAAA
TTCATTACTGACAAATACCAGAAAACTAATGTTGACAATATCTATGCTATAGGGGATGTGCTTCATGGAAAATTGGAA
CTTACTCCAACTGCAATTCAAGCGGGAAGACTATTGGCTGATAGACTATTTGCTGGAGGAACTACTACAATGGATTTT
TATGATGTTCCTACTACTATCTTTACTCCTCTTGAGTATGGATGAGTAGGTTACTCAGAGGAAGATGCTAGGGAGGAA
TATGGTGACTTCATCAAAGTTTACCATACTTATTTCCAGCCATTAGAATGGAACTTTGCAAAATCAATCTATAAGGAG
AGGAATTGATACGTAAAAATTATAATCAACACTGCAGATAATGACAGAGTGATCGGGTTCCATATTCTCTGTCCTAAT
GCTGGGGAGATAACACAAGGAATTGCTATTGCCATCAAAGTAGGAGTCACAAAGCCTCAGTTAGATAACTGTGTTGGA
ATTCATCCTACAATTGCTGAAGAAATGACTAATCTACATATTGATAAAGCTGATAATCCAGATCCAATTAAATCGGAT
TGCTGATCTTAA*AGAGTCTGTGCATATTAGGAGTTGCTAAAATTTAGCCAACTACTAATAACTGCATGGAAAAATAT*
AGTCGGTAAAGGAGACAAATCAGTAAGCAATGTTTCAAGACAATATGCTTGGTCTTTAGAAGCATTAGCTTTATGAAG
TCACCAGTCTCAAGCCCATGTGGTAGGCGGTGATGGAACAGCTGAGGACTCTCTGAGTAGTAATGGATCCACCG

Figure S10. Nucleotide sequence of the chimeric eTR1-hTR1 SECIS construct. The ORF is shown in black and the 3’-UTR sequence is shown in grey and italic. The Sec-encoding UGA and the UAA stop codons are shown in red. The sequence corresponding to the core of the SECIS element is also shown in red.

eTR1-hTR3Secis

GACTATTCAGACACTCCACAAGAAGAATCCACTCATAGTTATGACTATGATCTCTTTGTAATCGGAGGTGGTTCTGGA
GGGCTTGCTTGTGCCAAGGCTGCTCAAGAAGCAGGAGCTAAAGTAGCAGTAGCTGATTTTGTAAAGCCAACTCCAAAG
GGAACAAAGTGGAAAGTAGGAGGAACATGAGTGAATGTTGGTTGAATCCCCAAAAAGCTGATGCACTACTCCGCATTG
TTAGGAAATTCTTATCACGACCAAGTTGAGAGCGGATGGGAGCATGAGAAACCTTCTCATGACTGGGGTAAAATGATT
ACCAATGTCAATAACCATATAAGAGGTATCAATTTTGGATACAAAGCAGATATGAGAAAGAGAGGTATAAAGTTTCAT
GAAAAGTTTGCATCCTTTGTCGATCCTCATACCGTACAACTAGTTGATAAGAAGGGCAAGACCGAAATGATTACTTCT
AATTATTTCGTAATTGCTACTGGAGGCAGACCTCTCTATCCTGATATTCCAGGAGCCAAAGAGCATGCAATTACTAGT
GATGATATTTTCTGGATGAAAGACAACCCTGGTAAAACCCTTGTGGTAGGTGCTTCCTATGTCGCGTTGGAATGAGCT
GGATTTTTACATCATTTTGGAAACGAAGTTTCAGTTTGTGTCCGATCAATCTTTTTGAGAGGTTTCGATCAAGATATG
GCGCAAAAGATTGCTAAAGACATGGAACTCAGCGGGATTAATTTCATTAGAGACTCTGTGCCTACCAAAATTGAGAAA
GACGAAGAGACTGGCAAGCTCACCTGATTTTTAACAGTAGGAGGCGAAGAAACTACCGTTGAAGTAGATACTGTTCTT
TTTGCAATTGGTAGATATGCTGTGACAGCTGATTTAAATCTAGGTAATGCTGGACTCATTGCTGAAAAGAATGGAAAA
TTCATTACTGACAAATACCAGAAAACTAATGTTGACAATATCTATGCTATAGGGGATGTGCTTCATGGAAAATTGGAA
CTTACTCCAACTGCAATTCAAGCGGGAAGACTATTGGCTGATAGACTATTTGCTGGAGGAACTACTACAATGGATTTT
TATGATGTTCCTACTACTATCTTTACTCCTCTTGAGTATGGATGAGTAGGTTACTCAGAGGAAGATGCTAGGGAGGAA
TATGGTGACTTCATCAAAGTTTACCATACTTATTTCCAGCCATTAGAATGGAACTTTGCAAAATCAATCTATAAGGAG
AGGAATTGATACGTAAAAATTATAATCAACACTGCAGATAATGACAGAGTGATCGGGTTCCATATTCTCTGTCCTAAT
GCTGGGGAGATAACACAAGGAATTGCTATTGCCATCAAAGTAGGAGTCACAAAGCCTCAGTTAGATAACTGTGTTGGA
ATTCATCCTACAATTGCTGAAGAAATGACTAATCTACATATTGATAAAGCTGATAATCCAGATCCAATTAAATCGGAT
TGCTGATCTTAA*AGAGTCTGTGCATATTAGGAGTTGCTAAAATTTAGCCAACTACTAATAACTGCATGGGAAAAATAT*
AGTCGGTAAAGGAGACAAATCAGTAAGCAATGTTTCAAGACAATATGCTTGGTCTTTAGAAGCATTAGCTTTGATGAC
GACCTGGGTGGAAACCTACCCTGTGGGCACCCATGTCCGAGCAGCTGAGGACTCTCTGAGTAGTAATGGATCCACCG

Figure S11. Nucleotide sequence of the chimeric eTR1-hTR3 SECIS construct. The ORF is shown in black and the 3’-UTR sequence is shown in grey and italic. The Sec-encoding UGA and the UAA stop codons are shown in red. The sequence corresponding to the core of the SECIS element is also shown in red.

eTR2-hTR3ORF+3'UTR

GCTGACTCTGAAATGAAGAGTAATGAGTCAGAAGAATATGAAGAGACTAAGCGTCGTTATGATTATGATCTCTTCGTC
ATTGGTGGAGGATCAGGAGGACTTGCTTGTGCTAAAGCAGCTCAAGAGTGTGGAGCAAAGGTAGCTGTAGCTGACTTT
GTCAAGCCCTCTCCTCATGGATATGGAGTTGTGACTTGGGGAGTTGGTGGAACTTGTGTCAATGTGGGATGAATACCA
AAGAAATTATTACATTATTCAGCAAATTTGGGAGAAGCTTATGTTGATAGAGCTAGTAGTGGATGGGACCATGAGAAG
CCAAAACACGATTGGGGTAAAATGATTTCTAATATTAATAATCATATTCGAGCTATTAATTTTAGCATCAAAACTGAT
TTGAGGAAGAGAGGAATAAAATTTTATGAAAAATTAGCTTCTTTTGCTGATCCACATACTATTCAACTTTTAAACAAG
AAAGGCAAGACAGAATTAGTGACAGCAAATCATATTGTTATTGCAACTGGGGGAAGGCCTCTCTACCCTGATATCCCT
GGAGCAAAGGAGTATGGTATTACAAGCGATGACATTTTCTGGCTGAAGAAAAATCCAGGTAAAACCTTGGTCATTGGC
GCATCTTATATTGCACTTGAATGAGCTGGATTTTTACATAGTTTTGGTAACGATGTTTCTGTGTGAGTAAGATCGGTC
TTTTTGCGGGGCTTTGATCAGGATATGGCTAATATGCTTGCCAAGGATATGGAAGAACATGGTGGAGTCAAATTCATT
AAAAATTCAATACCTACCAAAATCGAAAAAGATGAAGAAACAGGAAAGCTCATATGATATCTCACCTCTAGAGAAGAG
GAAATTACTATAGAAGTTGACACAGTTTTGTTTGCAATTGGTAGATATGCTGTTACAAAAGATCTAAACCTTGAAAAT
GCGGGTCTCAAAGTAGAATCAAACGGTAAATTCATTACAGATGAGTTTCAACAAACTAATGTGGAGAATATCTATGCT
ATCGGAGATGTGATTCATGGGAAATTAGAACTAACACCCACTGCAATTCAAACAGGTAAACTACTTGCAAGAAGATTG
TATGCTGGTGAAACCACAACTATGGACTTTTGTGATATTCCAACTACAATCTTCACTCCTTTAGAGTATGGATGAGTT
GGATACTCAGAAGAAGAAGCTAAGGAAAAATATGGAGACGCCATTAAGGTATATCATACTTACTTCAAGCCATTAGAG
TGGAACTATGCAAAATCAATTTATAAATATCGAAATTGATATGTTAAAGTAATTATAAACACTACAGAGAATGATCGG
GTAATTGGCTATCATTTATTGGCTCCAAATGCAGGAGAAATTACTCAAGGAATTGCAATTGCCATTAAGATTGGCCTT
ACTAAACACAAGTTAGATAACTGTGTTGGAATCCATCCAACTGTTGCAGAAGAAGTCGACGTAGTCAAGCTGCGCATC
TCCAAGCGCTCAGGCCTGGACCCCACGGTGACAGGCTGCTGAGGGTAA*GCGCCATCCCTGCAGGCCAGGGCACACGGT*
GCGCCCGCCGCCAGCTCCTCGGAGGCCAGACCCAGGATGGCTGCAGGCCAGGTTTGGGGGGCCTCAACCCTCTCCTGG
AGCGCCTGTGAGATGGTCAGCGTGGAGCGCAAGTGCTGGACAGGTGGCCCGTGTGCCCCACAGGGATGGCTCAGGGGA
CTGTCCACCTCACCCCTGCACCTCTCAGCCTCTGCCGCCGGGCACCCCCCCCCAGGCTCCTGGTGCCAGATGATGACG
ACCTGGGTGGAAACCTACCCTGTGGGCACCCATGTCCGAGCCCCCTGGCATTTCTGCAATGCAAATAAAGAGGGTACT
TTTTCTGAAGTGTG

Figure S12. Nucleotide sequence of the chimeric eTR2-hTR3 construct. The ORF is shown in black and the 3'-UTR sequence is shown in grey and italic. The Sec-encoding UGA and the UAA stop codons are shown in red. The sequence corresponding to the hTR3 ORF is underlined. The sequence corresponding to the core of the SECIS element is shown in red.

eTR2-hTR3Secis

GCTGACTCTGAAATGAAGAGTAATGAGTCAGAAGAATATGAAGAGACTAAGCGTCGTTATGATTATGATCTCTTCGTC
ATTGGTGGAGGATCAGGAGGACTTGCTTGTGCTAAAGCAGCTCAAGAGTGTGGAGCAAAGGTAGCTGTAGCTGACTTT
GTCAAGCCCTCTCCTCATGGATATGGAGTTGTGACTTGGGGAGTTGGTGGAACTTGTGTCAATGTGGGATGAATACCA
AAGAAATTATTACATTATTCAGCAAATTTGGGAGAAGCTTATGTTGATAGAGCTAGTAGTGGATGGGACCATGAGAAG
CCAAAACACGATTGGGGTAAAATGATTTCTAATATTAATAATCATATTCGAGCTATTAATTTTAGCATCAAAACTGAT
TTGAGGAAGAGAGGAATAAAATTTTATGAAAAATTAGCTTCTTTTGCTGATCCACATACTATTCAACTTTTAAACAAG
AAAGGCAAGACAGAATTAGTGACAGCAAATCATATTGTTATTGCAACTGGGGGAAGGCCTCTCTACCCTGATATCCCT
GGAGCAAAGGAGTATGGTATTACAAGCGATGACATTTTCTGGCTGAAGAAAAATCCAGGTAAAACCTTGGTCATTGGC
GCATCTTATATTGCACTTGAATGAGCTGGATTTTTACATAGTTTTGGTAACGATGTTTCTGTGTGAGTAAGATCGGTC
TTTTTGCGGGGCTTTGATCAGGATATGGCTAATATGCTTGCCAAGGATATGGAAGAACATGGTGGAGTCAAATTCATT
AAAAATTCAATACCTACCAAAATCGAAAAAGATGAAGAAACAGGAAAGCTCATATGATATCTCACCTCTAGAGAAGAG
GAAATTACTATAGAAGTTGACACAGTTTTGTTTGCAATTGGTAGATATGCTGTTACAAAAGATCTAAACCTTGAAAAT
GCGGGTCTCAAAGTAGAATCAAACGGTAAATTCATTACAGATGAGTTTCAACAAACTAATGTGGAGAATATCTATGCT
ATCGGAGATGTGATTCATGGGAAATTAGAACTAACACCCACTGCAATTCAAACAGGTAAACTACTTGCAAGAAGATTG
TATGCTGGTGAAACCACAACTATGGACTTTTGTGATATTCCAACTACAATCTTCACTCCTTTAGAGTATGGATGAGTT
GGATACTCAGAAGAAGAAGCTAAGGAAAAATATGGAGACGCCATTAAGGTATATCATACTTACTTCAAGCCATTAGAG
TGGAACTATGCAAAATCAATTTATAAATATCGAAATTGATATGTTAAAGTAATTATAAACACTACAGAGAATGATCGG
GTAATTGGCTATCATTTATTGGCTCCAAATGCAGGAGAAATTACTCAAGGAATTGCAATTGCCATTAAGATTGGCCTT
ACTAAACACAAGTTAGATAACTGTGTTGGAATCCATCCAACTGTTGCAGAAGAAGTAACGGATCTCAAGATTGATAAA
GCAATCAATCCTGATCCAGTCAAGACAGATTGTTGATCTTAG*AGCATCTGTACATATTAGTCATTGCTTTTACAAAGG*
CAATCACTAATAGCAGTACGGAATAATTAATCTGAAAAGGGGAGGACCCAGTAAGCTATAGATCATACTCAAAATGAG
CTATAGCTTTGATGACGACCTGGGTGGAAACCTACCCTGTGGCACCCATGTCCGAGAAGCTGGTAATTCCCTGAGCA
GCTTCAGCAATAATTG

Figure S13. Nucleotide sequence of the chimeric eTR2-hTR3 SECIS construct. The ORF is shown in black and the 3'-UTR sequence is shown in grey and italic. The Sec-encoding UGA and the UAG stop codons are shown in red. The sequence corresponding to the core of the SECIS element is also shown in red.

eTR2-2x3′UTR

GCTGACTCTGAAATGAAGAGTAATGAGTCAGAAGAATATGAAGAGACTAAGCGTCGTTATGATTATGATCTCTTCGTC
ATTGGTGGAGGATCAGGAGGACTTGCTTGTGCTAAAGCAGCTCAAGAGTGTGGAGCAAAGGTAGCTGTAGCTGACTTT
GTCAAGCCCTCTCCTCATGGATATGGAGTTGTGACTTGGGGAGTTGGTGGAACTTGTGTCAATGTGGGATGAATACCA
AAGAAATTATTACATTATTCAGCAAATTTGGGAGAAGCTTATGTTGATAGAGCTAGTAGTGGATGGGACCATGAGAAG
CCAAAACACGATTGGGGTAAAATGATTTCTAATATTAATAATCATATTCGAGCTATTAATTTTAGCATCAAAACTGAT
TTGAGGAAGAGAGGAATAAAATTTTATGAAAAATTAGCTTCTTTTGCTGATCCACATACTATTCAACTTTTAAACAAG
AAAGGCAAGACAGAATTAGTGACAGCAAATCATATTGTTATTGCAACTGGGGGAAGGCCTCTCTACCCTGATATCCCT
GGAGCAAAGGAGTATGGTATTACAAGCGATGACATTTTCTGGCTGAAGAAAAATCCAGGTAAAACCTTGGTCATTGGC
GCATCTTATATTGCACTTGAATGAGCTGGATTTTTACATAGTTTTGGTAACGATGTTTCTGTGTGAGTAAGATCGGTC
TTTTTGCGGGGCTTTGATCAGGATATGGCTAATATGCTTGCCAAGGATATGGAAGAACATGGTGGAGTCAAATTCATT
AAAAATTCAATACCTACCAAAATCGAAAAAGATGAAGAAACAGGAAAGCTCATATGATATCTCACCTCTAGAGAAGAG
GAAATTACTATAGAAGTTGACACAGTTTTGTTTGCAATTGGTAGATATGCTGTTACAAAAGATCTAAACCTTGAAAAT
GCGGGTCTCAAAGTAGAATCAAACGGTAAATTCATTACAGATGAGTTTCAACAAACTAATGTGGAGAATATCTATGCT
ATCGGAGATGTGATTCATGGGAAATTAGAACTAACACCCACTGCAATTCAAACAGGTAAACTACTTGCAAGAAGATTG
TATGCTGGTGAAACCACAACTATGGACTTTTGTGATATTCCAACTACAATCTTCACTCCTTTAGAGTATGGATGAGTT
GGATACTCAGAAGAAGAAGCTAAGGAAAAATATGGAGACGCCATTAAGGTATATCATACTTACTTCAAGCCATTAGAG
TGGAACTATGCAAAATCAATTTATAAATATCGAAATTGATATGTTAAAGTAATTATAAACACTACAGAGAATGATCGG
GTAATTGGCTATCATTTATTGGCTCCAAATGCAGGAGAAATTACTCAAGGAATTGCAATTGCCATTAAGATTGGCCTT
ACTAAACACAAGTTAGATAACTGTGTTGGAATCCATCCAACTGTTGCAGAAGAAGTAACGGATCTCAAGATTGATAAA
GCAATCAATCCTGATCCAGTCAAGACAGATTGTTGATCTTAG*AGCATCTGTACATATTAGTCATTGCTTTTACAAAGG*
CAATCACTAATAGCAGTACGGAATAATTAATCTGAAAAGGGGAGGACCCAGTAAGCTATAGATCATACTCAAAATGAG
CTATAGCTTTTAGCATCTGTACATATTAGTCATTGCTTTTACAAAGGCAATCACTAATAGCAGTACGGAATAATTAAT
CTGAAAAGGGGAGGACCCAGTAAGCTATAGATCATACTCAAAATGAGCTATAGCTTTTATGATACTTCTTTCCTTCAA
AAATATAAAGGAAACAAGTTGAAAAGCTGGTAATTCCCTGAGCAGCTTCAGCAATAATTG

Figure S14. Nucleotide sequence of a modified eTR2 3’-UTR construct. The ORF is shown in black and the 3’-UTR sequence is shown in grey and italic. The Sec-encoding UGA and the UAG stop codons are shown in red. The duplicated sequence in the 3’-UTR is underlined. The sequence corresponding to the core of the SECIS element is shown in red.

eTR2-hTR3d11Secis

GCTGACTCTGAAATGAAGAGTAATGAGTCAGAAGAATATGAAGAGACTAAGCGTCGTTATGATTATGATCTCTTCGTC
ATTGGTGGAGGATCAGGAGGACTTGCTTGTGCTAAAGCAGCTCAAGAGTGTGGAGCAAAGGTAGCTGTAGCTGACTTT
GTCAAGCCCTCTCCTCATGGATATGGAGTTGTGACTTGGGGAGTTGGTGGAACTTGTGTCAATGTGGGATGAATACCA
AAGAAATTATTACATTATTCAGCAAATTTGGGAGAAGCTTATGTTGATAGAGCTAGTAGTGGATGGGACCATGAGAAG
CCAAAACACGATTGGGGTAAAATGATTTCTAATATTAATAATCATATTCGAGCTATTAATTTTAGCATCAAAACTGAT
TTGAGGAAGAGAGGAATAAAATTTTATGAAAAATTAGCTTCTTTTGCTGATCCACATACTATTCAACTTTTAAACAAG
AAAGGCAAGACAGAATTAGTGACAGCAAATCATATTGTTATTGCAACTGGGGGAAGGCCTCTCTACCCTGATATCCCT
GGAGCAAAGGAGTATGGTATTACAAGCGATGACATTTTCTGGCTGAAGAAAAATCCAGGTAAAACCTTGGTCATTGGC
GCATCTTATATTGCACTTGAATAGCTGGATTTTTACATAGTTTTGGTAACGATGTTTCTGTGTGAGTAAGATCGGTCT
TTTTGCGGGGCTTTGATCAGGATATGGCTAATATGCTTGCCAAGGATATGGAAGAACATGGTGGAGTCAAATTCATTA
AAAATTCAATACCTACCAAAATCGAAAAAGATGAAGAAACAGGAAAGCTCATATGATATCTCACCTCTAGAGAAGAGG
AAATTACTATAGAAGTTGACACAGTTTTGTTTGCAATTGGTAGATATGCTGTTACAAAAGATCTAAACCTTGAAAATG
CGGGTCTCAAAGTAGAATCAAACGGTAAATTCATTACAGATGAGTTTCAACAAACTAATGTGGAGAATATCTATGCTA
TCGGAGATGTGATTCATGGGAAATTAGAACTAACACCCACTGCAATTCAAACAGGTAAACTACTTGCAAGAAGATTGT
ATGCTGGTGAAACCACAACTATGGACTTTTGTGATATTCCAACTACAATCTTCACTCCTTTAGAGTATGGATGAGTTG
GATACTCAGAAGAAGAAGCTAAGGAAAAATATGGAGACGCCATTAAGGTATATCATACTTACTTCAAGCCATTAGAGT
GGAACTATGCAAAATCAATTTATAAATATCGAAATTGATATGTTAAAGTAATTATAAACACTACAGAGAATGATCGGG
TAATTGGCTATCATTTATTGGCTCCAAATGCAGGAGAAATTACTCAAGGAATTGCAATTGCCATTAAGATTGGCCTTA
CTAAACACAAGTTAGATAACTGTGTTGGAATCCATCCAACTGTTGCAGAAGAAGTAACGGATCTCAAGATTGATAAAG
CAATCAATCCTGATCCAGTCAAGACAGATTGTTGATCTTAG*AGCATCTGTACATATTAGTCATTGCTTTTACAAAGGC*
AATCACTAATAGCAGTACGGAATAATTAATCTGAAAAGGGGAGGACCCAGTAAGCTATAGATCATACTCAAAATGAGC
TATAGCTTTGATGACGACCTGGGTGGAAACGCACCCATGTCCGAGAAGCTGGTAATTCCCTGAGCAGCTTCAGCAATA
ATTG

Figure S15. Nucleotide sequence of the chimeric eTR2-hTR3 Δ11 SECIS construct. The ORF is shown in black and the 3'-UTR sequence is shown in grey and italic. The Sec-encoding UGA and the UAG stop codons are shown in red. The sequence corresponding to the core of the SECIS element is shown in red.

eTR2-hTR3d8Secis

GCTGACTCTGAAATGAAGAGTAATGAGTCAGAAGAATATGAAGAGACTAAGCGTCGTTATGATTATGATCTCTTCGTC
ATTGGTGGAGGATCAGGAGGACTTGCTTGTGCTAAAGCAGCTCAAGAGTGTGGAGCAAAGGTAGCTGTAGCTGACTTT
GTCAAGCCCTCTCCTCATGGATATGGAGTTGTGACTTGGGGAGTTGGTGGAACTTGTGTCAATGTGGGATGAATACCA
AAGAAATTATTACATTATTCAGCAAATTTGGGAGAAGCTTATGTTGATAGAGCTAGTAGTGGATGGGACCATGAGAAG
CCAAAACACGATTGGGGTAAAATGATTTCTAATATTAATAATCATATTCGAGCTATTAATTTTAGCATCAAAACTGAT
TTGAGGAAGAGAGGAATAAAATTTTATGAAAAATTAGCTTCTTTTGCTGATCCACATACTATTCAACTTTTAAACAAG
AAAGGCAAGACAGAATTAGTGACAGCAAATCATATTGTTATTGCAACTGGGGGAAGGCCTCTCTACCCTGATATCCCT
GGAGCAAAGGAGTATGGTATTACAAGCGATGACATTTTCTGGCTGAAGAAAAATCCAGGTAAAACCTTGGTCATTGGC
GCATCTTATATTGCACTTGAATAGCTGGATTTTTACATAGTTTTGGTAACGATGTTTCTGTGTGAGTAAGATCGGTCT
TTTTGCGGGGCTTTGATCAGGATATGGCTAATATGCTTGCCAAGGATATGGAAGAACATGGTGGAGTCAAATTCATTA
AAAATTCAATACCTACCAAAATCGAAAAAGATGAAGAAACAGGAAAGCTCATATGATATCTCACCTCTAGAGAAGAGG
AAATTACTATAGAAGTTGACACAGTTTTGTTTGCAATTGGTAGATATGCTGTTACAAAAGATCTAAACCTTGAAAATG
CGGGTCTCAAAGTAGAATCAAACGGTAAATTCATTACAGATGAGTTTCAACAAACTAATGTGGAGAATATCTATGCTA
TCGGAGATGTGATTCATGGGAAATTAGAACTAACACCCACTGCAATTCAAACAGGTAAACTACTTGCAAGAAGATTGT
ATGCTGGTGAAACCACAACTATGGACTTTTGTGATATTCCAACTACAATCTTCACTCCTTTAGAGTATGGATGAGTTG
GATACTCAGAAGAAGAAGCTAAGGAAAAATATGGAGACGCCATTAAGGTATATCATACTTACTTCAAGCCATTAGAGT
GGAACTATGCAAAATCAATTTATAAATATCGAAATTGATATGTTAAAGTAATTATAAACACTACAGAGAATGATCGGG
TAATTGGCTATCATTTATTGGCTCCAAATGCAGGAGAAATTACTCAAGGAATTGCAATTGCCATTAAGATTGGCCTTA
CTAAACACAAGTTAGATAACTGTGTTGGAATCCATCCAACTGTTGCAGAAGAAGTAACGGATCTCAAGATTGATAAAG
CAATCAATCCTGATCCAGTCAAGACAGATTGTTGATCTTAG*AGCATCTGTACATATTAGTCATTGCTTTTACAAAGGC*
AATCACTAATAGCAGTACGGAATAATTAATCTGAAAAGGGGAGGACCCAGTAAGCTATAGATCATACTCAAAATGAGC
TATAGCTTTGATGACGACCTGGGTGGAAACTGGGCACCCATGTCCGAGAAGCTGGTAATTCCCTGAGCAGCTTCAGCA
ATAATTG

Figure S16. Nucleotide sequence of the chimeric eTR2-hTR3 Δ8 SECIS construct. The ORF is shown in black and the 3'-UTR sequence is shown in grey and italic. The Sec-encoding UGA and the UAG stop codons are shown in red. The sequence corresponding to the core of the SECIS element is shown in red.

eTR2-hSelP3′UTR

GCTGACTCTGAAATGAAGAGTAATGAGTCAGAAGAATATGAAGAGACTAAGCGTCGTTATGATTATGATCTCTTCGTC
ATTGGTGGAGGATCAGGAGGACTTGCTTGTGCTAAAGCAGCTCAAGAGTGTGGAGCAAAGGTAGCTGTAGCTGACTTT
GTCAAGCCCTCTCCTCATGGATATGGAGTTGTGACTTGGGGAGTTGGTGGAACTTGTGTCAATGTGGGATGAATACCA
AAGAAATTATTACATTATTCAGCAAATTTGGGAGAAGCTTATGTTGATAGAGCTAGTAGTGGATGGGACCATGAGAAG
CCAAAACACGATTGGGGTAAAATGATTTCTAATATTAATAATCATATTCGAGCTATTAATTTTAGCATCAAAACTGAT
TTGAGGAAGAGAGGAATAAAATTTTATGAAAAATTAGCTTCTTTTGCTGATCCACATACTATTCAACTTTTAAACAAG
AAAGGCAAGACAGAATTAGTGACAGCAAATCATATTGTTATTGCAACTGGGGGAAGGCCTCTCTACCCTGATATCCCT
GGAGCAAAGGAGTATGGTATTACAAGCGATGACATTTTCTGGCTGAAGAAAAATCCAGGTAAAACCTTGGTCATTGGC
GCATCTTATATTGCACTTGAATGAGCTGGATTTTTACATAGTTTTGGTAACGATGTTTCTGTGTGAGTAAGATCGGTC
TTTTTGCGGGGCTTTGATCAGGATATGGCTAATATGCTTGCCAAGGATATGGAAGAACATGGTGGAGTCAAATTCATT
AAAAATTCAATACCTACCAAAATCGAAAAAGATGAAGAAACAGGAAAGCTCATATGATATCTCACCTCTAGAGAAGAG
GAAATTACTATAGAAGTTGACACAGTTTTGTTTGCAATTGGTAGATATGCTGTTACAAAAGATCTAAACCTTGAAAAT
GCGGGTCTCAAAGTAGAATCAAACGGTAAATTCATTACAGATGAGTTTCAACAAACTAATGTGGAGAATATCTATGCT
ATCGGAGATGTGATTCATGGGAAATTAGAACTAACACCCACTGCAATTCAAACAGGTAAACTACTTGCAAGAAGATTG
TATGCTGGTGAAACCACAACTATGGACTTTTGTGATATTCCAACTACAATCTTCACTCCTTTAGAGTATGGATGAGTT
GGATACTCAGAAGAAGAAGCTAAGGAAAAATATGGAGACGCCATTAAGGTATATCATACTTACTTCAAGCCATTAGAG
TGGAACTATGCAAAATCAATTTATAAATATCGAAATTGATATGTTAAAGTAATTATAAACACTACAGAGAATGATCGG
GTAATTGGCTATCATTTATTGGCTCCAAATGCAGGAGAAATTACTCAAGGAATTGCAATTGCCATTAAGATTGGCCTT
ACTAAACACAAGTTAGATAACTGTGTTGGAATCCATCCAACTGTTGCAGAAGAAGTAACGGATCTCAAGATTGATAAA
GCAATCAATCCTGATCCAGTCAAGACAGATTGTTGATCTTAGGTCGAC*TAGGACATACTCCCCAATTTAGTCTAGACA*
CAATTTCATTTCCAGCATTTTTATAAACTACCAAATTAGTGAACCAAAAATAGAAATTAGATTTGTGCAAACATGGAG
AAATCTACTGAATTGGCTTCCAGATTTTAAATTTTATGTCATAGAAATATTGACTCAAACCATATTTTTTATGATGGA
GCAACTGAAAGGTGATTGCAGCTTTTGGTTAATATGTCTTTTTTTTTCTTTTTCCAGTGTTCTATTTGCTTTAATGAG
AATAGAAACGTAAACTATGACCTAGGGGTTTCTGTTGGATAATTAGCAGTTTAGAATGGAGGAAGAACAACAAAGACA
TGCTTTCCATTTTTTTCTTTACTTATCTCTCAAAACAATATTACTTTGTCTTTTCAATCTTCTACTTTTAACTAATAA
AATAAGTGGATTTTGTATTTTAAGATCCAGAAATACTTAACACGTGAATATTTTGCTAAAAAAGCATATATAACTATT
TTAAATATCCATTTATCTTTTGTATATCTAAGACTCATCCTGATTTTTACTATCACACATGAATAAAGCCTTTGTATC
TTTCTTTCTCTAATGTTGTATCATACTCTTCTAAAACTTGAGTGGCTGTCTTAAAAGATATAAGGGGAAAGATAATAT
TGTCTGTCTCTATATTGCTTAGTAAGTATTTCCATAGTCAATGATGGTTTAATAGGTAAACCAAACCCTATAAACCTG
ACCTCCTTTATGGTTAATACTATTAAGCAAGAATGCAGTACAGAATTGGATACAGTACGGA

Figure S17. Nucleotide sequence of the chimeric eTR2-hSelP 3-UTR construct. The ORF is shown in black and the 3’-UTR sequence is shown in grey and italic. The Sec-encoding UGA and the UAG stop codons are shown in red. The sequences corresponding to the core of the SECIS elements are shown in red.

eSelW

GAACAAAAACTTATTTCTGAAGAAGATCTGAAAATTCAGTTCTGTGGAGGCTGATCATACCGTCCCAAAGCTGTCTAT
GTCCAGAAGGAGGTAGAAAAGATCTTTGGTGAAAAGTTAGCTGTTATTTTTAAGAAAGACTTGAAGGTAACTGGGAAT
TTCGAAATAATCCTCTTTAATCAGAAGACGGGTGAGTCGAAGTTGGTTCATAGTAAGAAGAATGGTGGTGGCTTCGTG
AAGGAGGATAATTTTGATGAGTTTAAAGAAAAACTCGCCGAATTCTGATCATCTTAA*TCATCAACATAGCCTCTTTCT*
GTTTCAACAGATATATTGCATATTTTAATGATCACCTGTCTCTTCAAACCCATAAGAGCAGGAGTGAACCCAATATGA
ATATTTCAGTTGGATTTATACGCTTCTAAGCGTAGG

Figure S18. Nucleotide sequence of the eSelW construct. The ORF is shown in black and the 3'-UTR sequence is shown in grey and italic. The Sec and Cys-encoding UGA and the UAA stop codons are shown in red. The sequence corresponding to the core of the SECIS element is also shown in red. The sequence corresponding to the N-terminal myc-tag is shown in bold and is underlined.

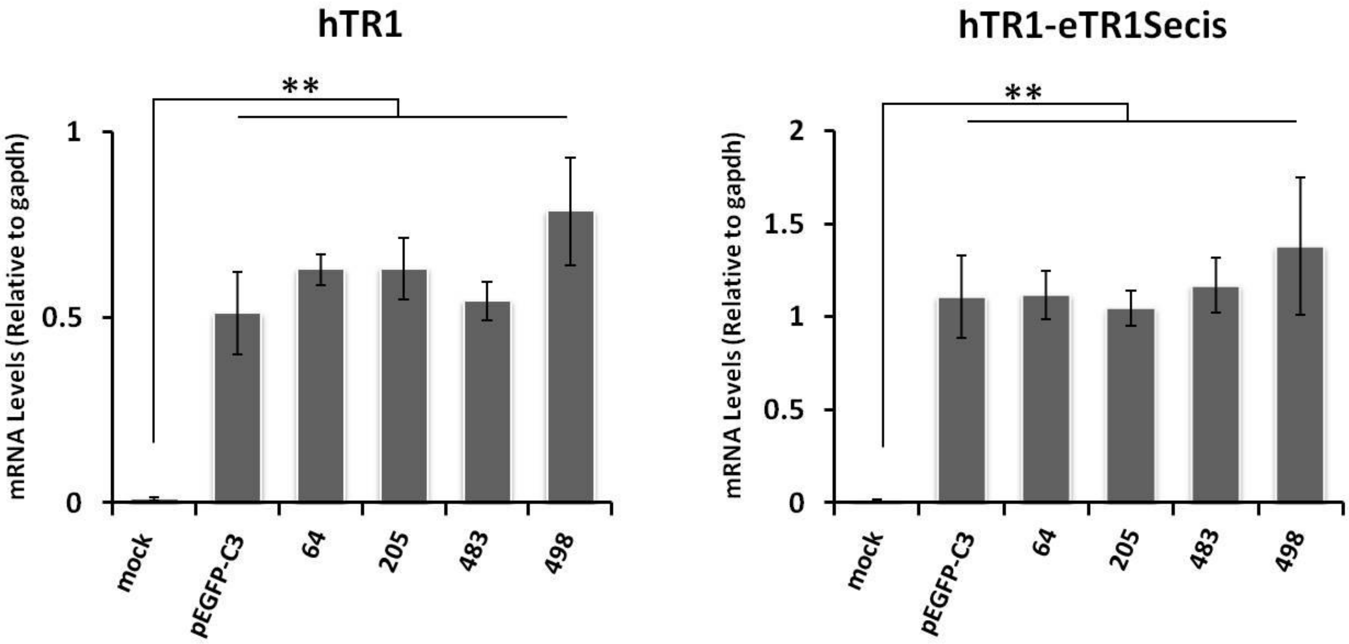

Figure S19. Relative abundance of hTR1 and hTR1-eTR1 SECIS mRNAs. Total mRNA was isolated from HEK 293 cells transfected with the indicated constructs and used for cDNA synthesis and qPCR analysis. RNA from untransfected HEK cells served as a mock control. Levels of each target mRNA (EGFP for all constructs) relative to GAPDH are shown and the results represent mean value of three replicates ±SD. The difference between mock and plasmid transfected cells was statistically significant with the p-value $p<0.01$ for *hTR1* and *hTR1-eTR1Secis* (calculated by Student's t test, one-tailed). The double asterisk (**) indicates significant difference in proportions at the 1% level.

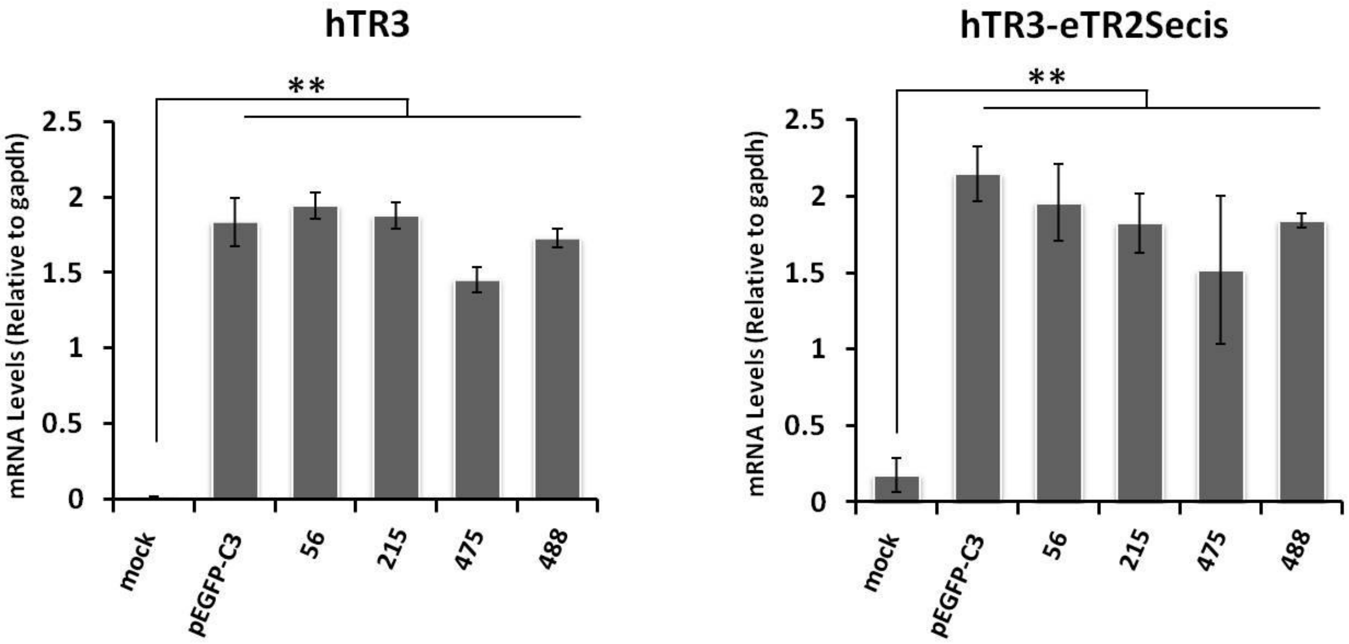

Figure S20. Relative abundance of hTR3 and hTR3-eTR2 SECIS mRNAs. Total mRNA was isolated from HEK 293 cells transfected with the indicated constructs and used for cDNA synthesis and qPCR analysis. RNA from untransfected HEK cells served as a mock control. Levels of each target mRNA (EGFP for all constructs) relative to GAPDH are shown and the results represent mean value of three replicates $\pm$SD. The difference between mock and plasmid transfected cells was statistically significant with the p-value $p<0.01$ for *hTR3* and *hTR3-eTR2Secis* (calculated by Student's t test, one-tailed). The double asterisk (**) indicates significant difference in proportions at the 1% level.

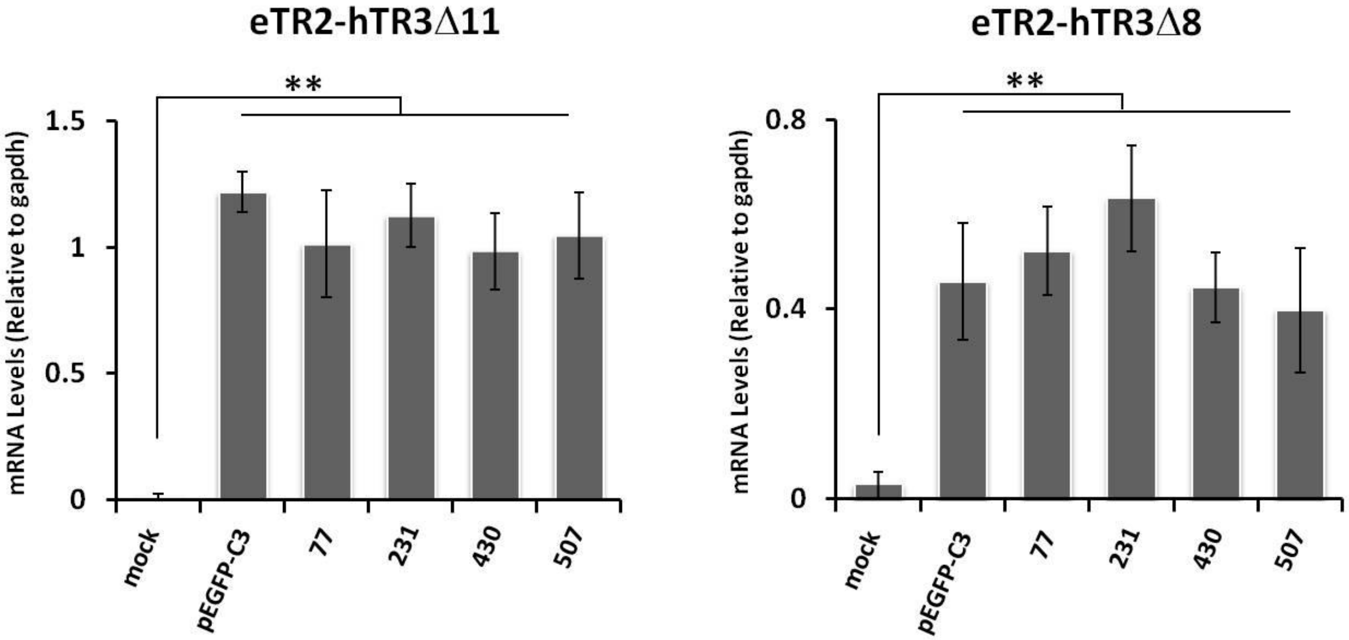

Figure S21. Relative abundance of eTR2-hTR3 Δ11 SECIS and eTR2-hTR3 Δ8 SECIS mRNAs. Total mRNA was isolated from HEK 293 cells transfected with the indicated constructs and used for cDNA synthesis and qPCR analysis. RNA from untransfected HEK cells served as a mock control. Levels of each target mRNA (EGFP for all constructs) relative to GAPDH are shown and the results represent mean value of three replicates +SD. The difference between mock and plasmid transfected cells was statistically significant with the p-value $p<0.01$ for *eTR2-hTR3Δ11* and *eTR2-hTR3Δ8* (calculated by Student's t test, one-tailed). The double asterisk (**) indicates significant difference in proportions at the 1% level.

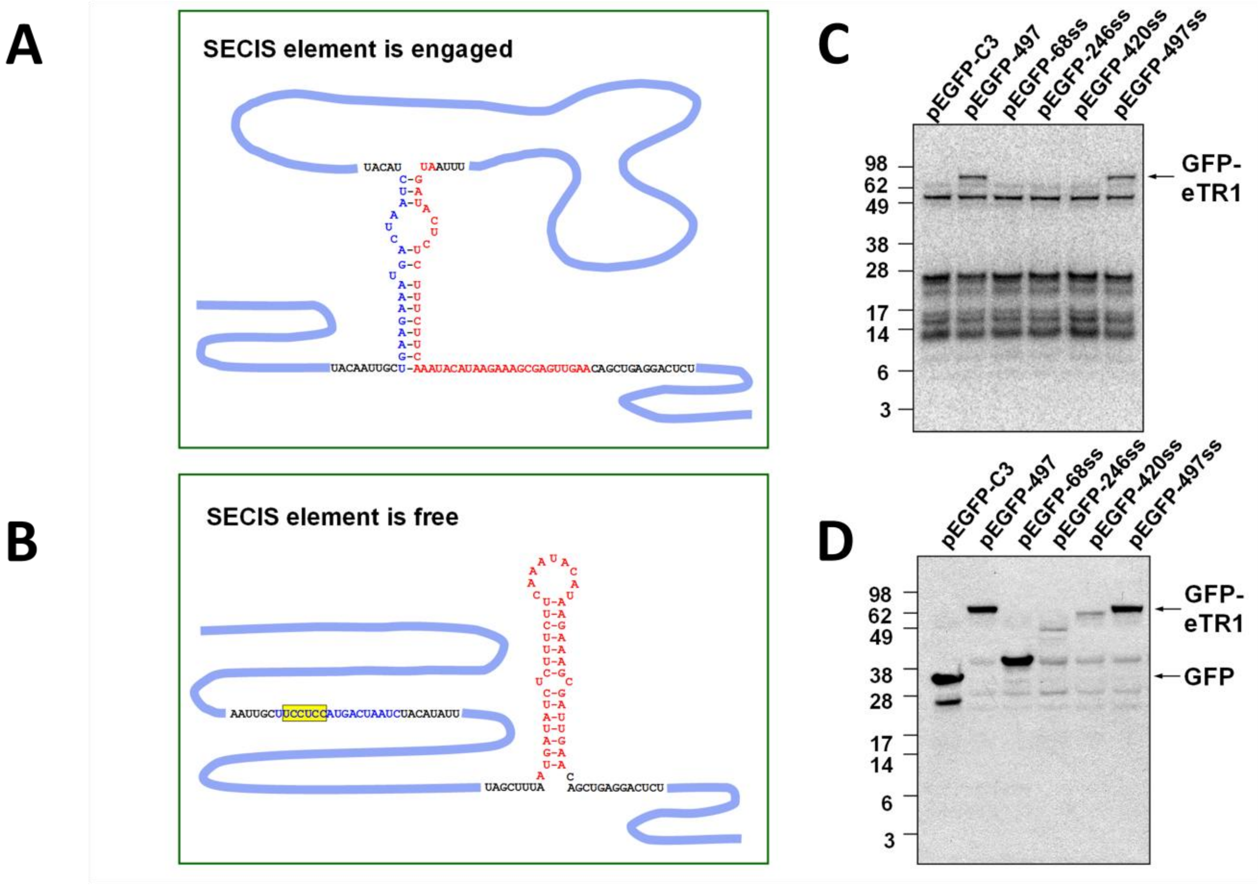

Figure S22. Mutation of a segment complementary to a part of the SECIS does not influence the coding function of UGA codon. (A) Nucleotide sequence (shown in blue) complementary to the 5'-sequence of the SECIS element was identified 47 bp upstream of the UGA codon. This sequence could possibly pair with the corresponding part of the SECIS element, thus disrupting the stem-loop structure. The SECIS element is shown in red. (B) Six nucleotides (highlighted in yellow) were mutated to prevent possible pairing. No changes in Sec incorporation were observed. (C) Expression of EGFP-eTR1 in HEK 293 cells. Cells were transfected with the vector (pEGFP-C3), the pEGFP-eTR1 construct containing a single UGA codon at the natural Sec position 497 (pEGFP-e479), or constructs with single in-frame UGA in which the number indicates the amino acid residue for which the UGA codon is retained or introduced, plus mutation of six nucleotides corresponding to 1426-1431 bp in eTR1 ORF: pEGFP-68ss, pEGFP-246ss, pEGFP-420ss and pEGFP-497ss. Cells were analyzed as described in the legend of Figure 2 in the text. *Arrow* shows the position of the GFP-eTR1 fusion selenoprotein. (D) Western blotting of samples shown in A with anti-GFP antibodies. *Arrows* show the positions of EGFP and full-size EGFP-eTR1. Molecular masses of protein standards (in kDa) are shown on the *left.*

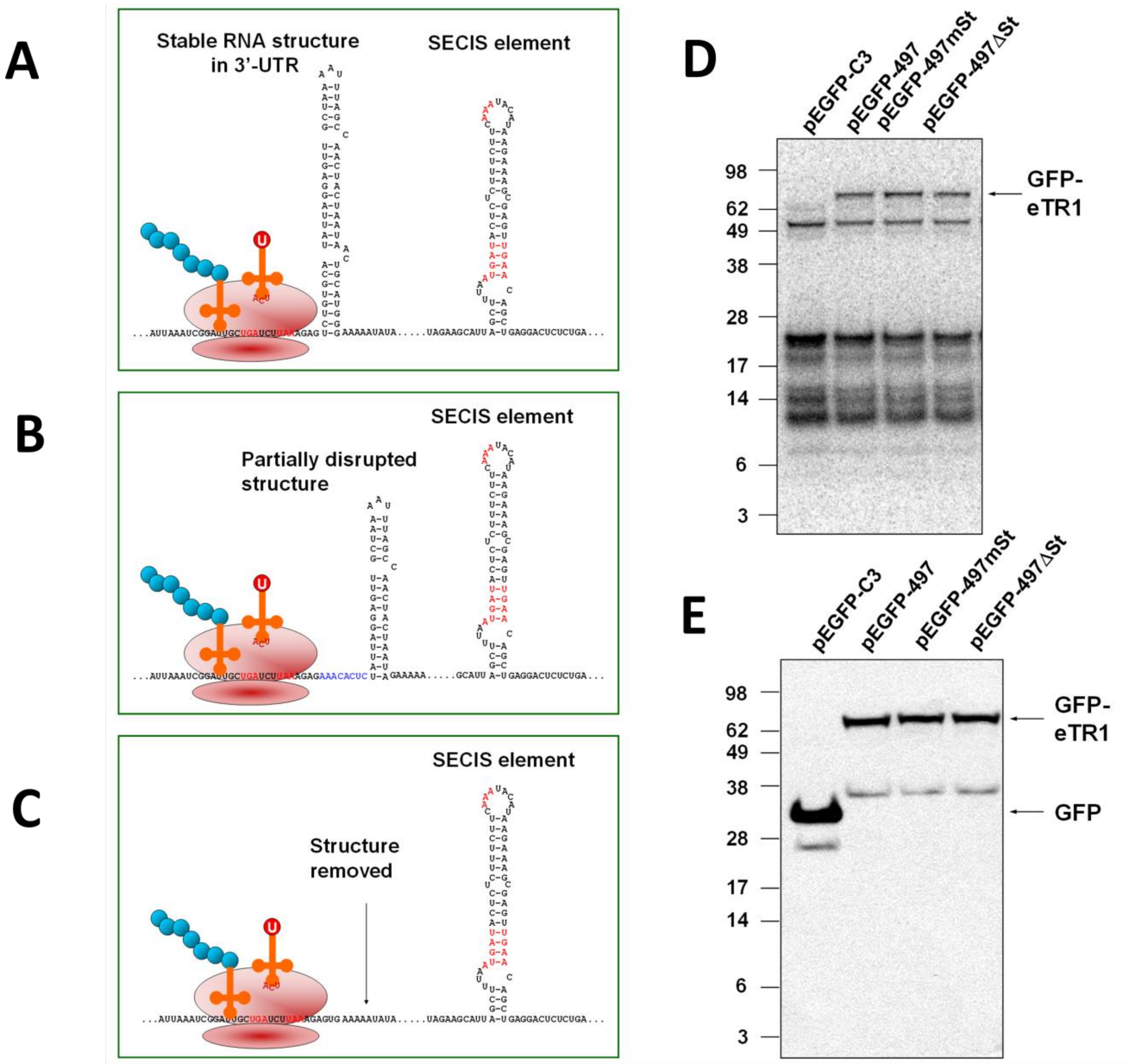

Figure S23. Testing the role of the mRNA structure in the 3'-UTR on Sec insertion in eTR1. (A) A stable stem-loop structure was identified 10 nt downstream of the Sec UGA codon. It was examined for the role in Sec insertion. The SECIS core, stop codon and UGA Sec codon are shown in red. (B) The structure was partially disrupted by mutating eight nucleotides (shown in blue). (C) The structure was completely removed. Both mutations did not affect the function of the Sec codon. (D) Expression of EGFP-eTR1 in HEK 293 cells. Cells were transfected with the vector (pEGFP-C3), a pEGFP-eTR1 construct containing a single UGA codon at the natural Sec position 497 (pEGFP-e479) or pEGFP-e479 constructs with either mutations of eight nucleotides corresponding to 1502-1509 bp in 3'-UTR of eTR1 mRNA (pEGFP-e497mSt) or deletion of 52 nucleotides corresponding to 1502-1554 bp in 3'-UTR of eTR1 mRNA (pEGFP-e497DSt). *Arrow* shows the position of the EGFP-eTR1 fusion selenoprotein. (E) Western blotting of samples shown in d with anti-GFP antibodies. *Arrows* show the positions of EGFP and full-size EGFP-eTR1. Molecular masses of protein standards (in kDa) are shown on the *left*.

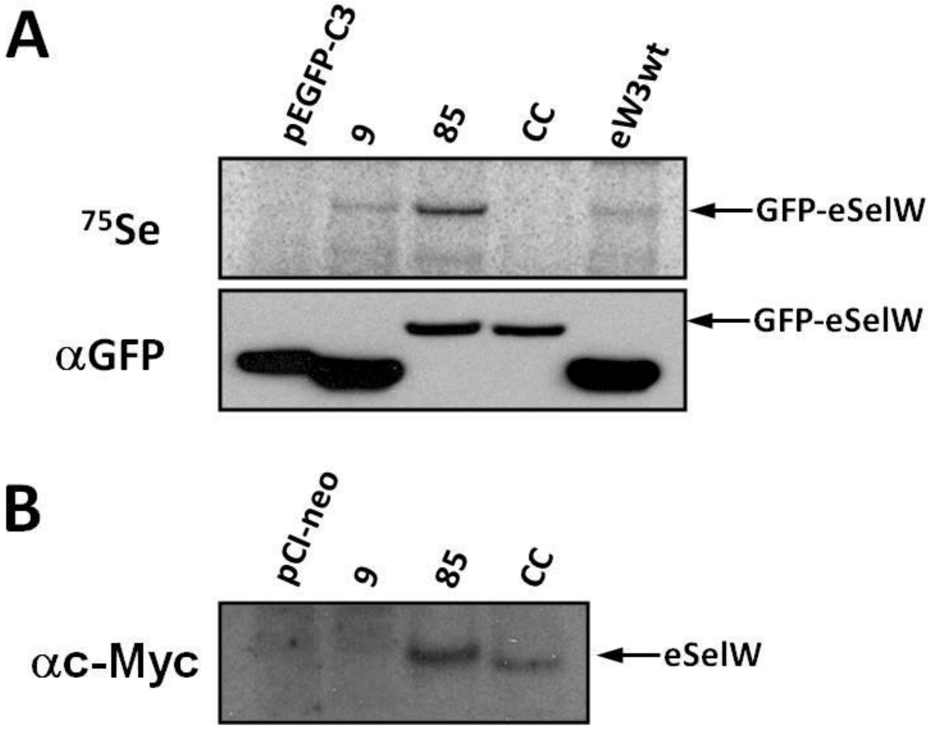

Figure S24. Position-dependent Sec insertion in eSelW. (A) Expression of EGFP-eSelW in HEK 293 cells. Sec insertion in eSelW with its natural SECIS element. (B) Expression of c-Myc-eSelW in HEK 293 cells revealed by Western blotting against c-Myc. SelW constructs with single in-frame UGA codons (at positions *9* or *85*) were used to examine Sec insertion (designated as 9 and 85, respectively, in the figure). The construct in which both UGA codons were replaced with cysteine codons (U9C, U85C) was used as a positive control (designated *CC* in the figure). The WT eSelW construct with two in-frame UGA codons was used as a negative control (*eWwt*). The experiment was carried out as described in the legend to Figure 2.

**Target sequence**			**PCR product length, bp**
EGFP	forward	GCATCGACTTCAAGGAGGAC	178
	reverse	GTGTTCTGCTGGTAGTGGT	
GAPDH	forward	TGCACCACCAACTGCTTAGC	87
	reverse	GGCATGGACTGTGGTCATGAG	

Table S1. Oligonucleotide primers used for qPCR.
